# Inter- and intra-individual variations in seasonal and daily stabilities of the human gut microbiota in Japanese

**DOI:** 10.1007/s00203-015-1125-0

**Published:** 2015-06-12

**Authors:** Takayoshi Hisada, Kaori Endoh, Kiyonori Kuriki

**Affiliations:** Laboratory of Public Health, Graduate School of Nutritional and Environmental Sciences, University of Shizuoka, 52-1 Yada, Suruga-ku, Shizuoka 422-8526 Japan; TechnoSuruga Laboratory Co., Ltd., 330 Nagasaki, Shimizu-ku, Shizuoka 424-0065 Japan

**Keywords:** Human gut microbiota, Inter-individual variations, Intra-individual variations, Next-generation sequencing, Seasonal and daily stabilities

## Abstract

Relationships between human gut microbiota, dietary habits, and health/diseases are the subject of epidemiological and clinical studies. However, the temporal stability and variability of the bacterial community in fecal samples remain unclear. In this study, middle-aged Japanese male and female volunteers (*n* = 5 each) without disease were recruited from the Sakura Diet Study. Fecal samples and lifestyle information were collected in every quarter and at each defecation for 7 continuous days. Next-generation sequencing of 16S rDNA and hierarchical clustering showed no time trend and intra-individual differences in both fecal sample sets. Significant inter-individual variations in seasonal and daily fecal sample sets were detected for 24 and 23 out of 39 selected dominant genera (>0.1 % of the total human gut microbiota; occupation rate >85 %), respectively. Intra- to inter-individual variance ratios in 26 and 35 genera were significantly <1.0 for seasonal and daily stabilities. Seasonal variation in fermented milk consumption might be associated with *Bifidobacterium* composition, but not with *Lactobacillus*. For most of the dominant genera in the human gut microbiota, inter-individual variations were significantly larger than intra-individual variations. Further studies are warranted to determine the contributions of human gut microbiota to nutritional metabolism, health promotion, and prevention/development of diseases.

**Electronic supplementary material:**

The online version of this article (doi:10.1007/s00203-015-1125-0) contains supplementary material, which is available to authorized users.

## Introduction

The human intestinal bacterial community is diverse (more than 1000 species) and varies between individuals (Turnbaugh et al. 2009; Lahti et al. 2014). Human gut microbiota is thought to play important roles in association with dietary habits and diseases such as obesity (Ley et al. 2006), inflammatory bowel disease (Walters et al. 2014), and cancer (Schwabe and Jobin 2013). Human intestinal microbiota has been assessed by using terminal restriction fragment length polymorphism analysis (Nagashima et al. 2003); however, identifying each bacterial species is difficult, as it requires the sequencing of all corresponding fragments in a sample. Recently, the use of next-generation sequencing (NGS) led to new findings on specific genera and species of human intestinal bacteria (Shokralla et al. 2012; Quail et al. 2012) with a relatively higher sensitivity and accuracy with respect to bacterial identification.

In recent case–control studies, significant differences in some intestinal bacteria were found between healthy controls and patients with colorectal cancer (Wang et al. 2012; Wu et al. 2013; Ahn et al. 2013). However, consistent findings between studies have not been reported regarding the risk of colorectal cancer. The subject-specific microbiota is thought to be stable for a long period (Rajilić-Stojanović et al. 2012; Martínez et al. 2013; Faith et al. 2013), whereas the composition of each human intestinal bacterium within the microbiota has been demonstrated to change rapidly with a diet rich in fermented milk (Veiga et al. 2014) and animal-based products (David et al. 2014). Thus, the temporal stability and variance remain unclear, rendering bacterial analysis in epidemiological and clinical studies.

Using two sets of seasonal and daily fecal samples from middle-aged Japanese male and female volunteers (*n* = 5 each) without disease recruited from the Sakura Diet Study, seasonal and daily stabilities were analyzed by NGS of 16S rDNA and hierarchical clustering. We then examined the relative contribution of the inter- and intra-individual variance to each human intestinal bacterium.

## Materials and methods

### Study subjects

Fecal samples were collected from 10 middle-aged Japanese subjects (five men and five women) from January 2013 to March 2014. The subjects were systematically recruited (based on age, gender, residence, and non-severe diseases such as heart and cerebrovascular diseases, and cancer) as volunteers from participants in the Sakura Diet Study in Shizuoka, located in central Japan. They had lived in the area for at least 1 year. Briefly, we orally explained to them the purpose of the study and obtained their signed informed consent for participation in this study. Lifestyle information, including dietary habits, was collected using a scientifically validated questionnaire (Tokudome et al. 2005). During 1-year interval between the first and the second administration of the food frequency questionnaires, the following samples were systematically collected each season: a 3-day dietary record, blood, urine, saliva, feces, and green tea samples as well as blood pressure, height, and body weight. The study was approved by the Ethics Committee, University of Shizuoka (No. 24-24).

### Questionnaires on defecation and collection of fecal samples

We asked the subjects to collect fecal samples and fill out questionnaires on defecation times in every quarter (four times, representing seasonal samples), and at all times of defecation on 7 continuous days after the fourth season (more than three times in 1 week, considering ones with constipation). For both sets of samples, soon after defecation, each fecal sample was individually suspended by the study subjects in 100 mM Tris–HCl (pH 9), 40 mM EDTA, 4 M guanidine thiocyanate (protein denaturant to inhibit bacterial growth), and 0.001 % bromothymol, as previously described (Fukuda and Fujita 2014; Shiozaki et al. 2014). To clarify inter- and intra-individual variations of human gut microbiota, we collected seasonal and daily fecal sample sets from the same subjects.

The questionnaire on defecation included questions on defecation clock time, stool volume, stool shape/consistency, and stool color, along with questions regarding dietary intake of fermented milk (i.e., yoghurt and fermented milk beverages), antibiotics, other medication, probiotics, and prebiotics within 1 week or 1 day before fecal collection. Stool volume was recorded in terms of the number (e.g., 0.5, 1 and 2) of Japanese standard chicken egg size “S” (i.e., 46–52 g). Color was selected from the following categorical variables; “yellow,” “green ocher,” “brown,” “blackish brown,” or “slightly blackish,” and shape/consistency was selected from the following categorical variables: “watery,” “muddy,” “soft,” “banana shape,” “hard,” or “very hard” (Nakamura and Oku 2002).

### DNA extraction from fecal samples

Fecal solids in the suspension were broken down by using FastPrep 24 Instrument (MP Biomedicals, Santa Ana, CA, USA) with zirconia beads at 5 m/s for 2 min. Bacterial DNA extraction from 200 μL of the suspension was performed by using a Magtration System 12GC (Precision System Science, Japan), with MagDEA DNA 200 (Precision System Science) as a reagent for the automatic nucleic acid extraction.

### NGS analysis of bacterial community structure in feces

In this study, a series of representative bacteria in the human gut microbiota was analyzed by previously described NGS of 16S rDNA methods (Takahashi et al. 2014) using the following primers (for the V3–V4 region of 16S rDNA of prokaryotes): forward primer 5′-AATGATACGGCGACCACCGAGATCTACACXXXXXXXXACACTCTTTCCCTACACGACGCTCTTCCGATCTCCTACGGGNBGCASCAG-3′, where Xs represent the sample-specific 8-bp barcode sequences (CTCTCTAT, TATCCTCT, GTAAGGAG, ACTGCATA, AAGGAGTA, CTAAGCCT, CGTCTAAT, TCTCTCCG, TCGACTAG, and TTCTAGCT) and reverse primer 5′-CAAGCAGAAGACGGCATACGAGATZZZZZZZZGTGACTGGAGTTCAGACGTGTGCTCTTCCGATCTGACTACNVGGGTATCTAATCC-3′, where Zs represent the sample-specific 8-bp barcode sequences (TCGCCTTA, CTAGTACG, TTCTGCCT, GCTCAGGA, AGGAGTCC, CATGCCTA, GTAGAGAG, CAGCCTCG, TGCCTCTT, TCCTCTAC, TCATGAGC, and CCTGAGAT); the underlined sequences represent the PCR primer region (Pro341F and Pro805R). Sequencing was conducted using a paired-end and modified to 2 × 300-bp cycle run on an Illumina MiSeq sequencing system (Illumina, San Diego, CA, USA) and MiSeq Reagent Kit version 3 (600 Cycle) chemistry. Paired-end sequencing with read lengths of 301 bp was performed. After demultiplexing, a clear overlap in the paired-end reads was observed. The method of quality filtering of sequences was as follows: only reads that had quality value (QV) scores of ≥20 for more than 99 % of the sequence were extracted for further analysis.

### 16S rDNA-based taxonomic analysis

Bacterial identification from sequences was performed using the Metagenome@KIN analysis software (World Fusion, Japan) and the TechnoSuruga Lab Microbial Identification database DB-BA 9.0 (TechnoSuruga Laboratory, Japan). Regarding the composition (%) of human intestinal bacteria, we focused on genera representing >0.1 % of the total human gut microbiota, considering the measurement precision, which was approximately >85 % in this study. Based on the analysis of a series of 20 pooled standard samples (i.e., a mixture of our study samples) measured within one run (for 2 days), intra-assay coefficients of variation were <5.0, <10.0, and <15.0 % for 6, 7, and 9 genera, respectively, out of the 22 selected human intestinal bacteria (>0.2 % of the total human gut microbiota), except for *Odoribacter.* Inter-assay coefficients of variation based on replicate analyses of a total of 60 pooled standard samples over three runs were <7.0 % (<5.0 % for 13 genera out of these), except for *Bifidobacterium* and *Akkermansia*. For the remaining genera (including *Collinsella*, 0.1–0.2 % of the total human gut microbiota), however, the intra- and inter-assay coefficients were >15.0, and 2.3–14.4 %, respectively.

### Statistical analyses

Considering the measurement precision, in the stability analyses, hierarchical clustering for the selected 17 human intestinal bacterium (>1.0 % of the total human gut microbiota) was performed using GeneMaths software (Applied Maths, Belgium) to obtain an overview of the similarity between 16S rDNA genomic profiles in the subjects. For clustering, the similarity between the profiles was calculated using Pearson’s correlation coefficient (*r*_p_), while linkage was calculated using the Ward method. Each variation in the profiles was visualized by different lengths of branches (i.e., *r*_p_) in the clustering tree for seasonal and daily fecal sample sets. For mean values of each human intestinal bacterium over four seasons (*v*__four seasons_ %), *r*_p_ (only for >1.0 % of the total human gut microbiota, considering *r*_p_ based on hierarchical clustering analysis) and Spearman’s correlation coefficients (*r*_s_) are shown with each genus for 1–3 continuous days (*v*__1d_, *v*__2d_, and *v*__3d_ %, in the order). However, regarding *r*_p_ and *r*_s_ for daily fecal sample set, the two mean values of 1–3 and 7 days could not be used as independent variables.

Cochran’s *Q*-test was used to assess changes in fermented milk intake. For the selected 39 human intestinal bacteria, the inter- and intra-individual differences from each composition of the human intestinal microbiota were analyzed by one-way repeated measures ANOVA. Using a single imputation method, each mean value was used to replace missing values for subjects who could not provide their fecal samples because of constipation. In case of subjects with two or more defecations in a day, mean value of all defecations in the day was used. Friedman test, as a nonparametric method, was also performed, considering the small sample size, i.e., 40 seasonal and 72 daily fecal samples obtained from the 10 subjects. The total variance was partitioned by ANOVA into the following two sources: (1) inter-individual variance in the subject’s stationary composition and (2) residual variance, which measured intra-individual variance in either the seasonal or daily fecal sample set (Ogawa et al. 1999). We also calculated the number of days needed to estimate the true value with 95 % confidence intervals within 10 and 20 % of their true mean (Beaton et al. 1979). These analyses were performed with SPSS version 18 (IBM Corporation, Chicago, IL, USA).

## Results

### Subject characteristics

Means [standard deviation (SD)] of age and BMI were 37.2 (2.5) and 38.2 (10.0) years and 26.4 (3.5) and 23.1 (1.6) kg/m^2^ in men and women, respectively. Fermented milk intake and defecation conditions of each subject are presented in Table 1. Significant seasonal variation was found for fermented milk intake (times/week) (*p* = 0.022). Probiotics, prebiotics, or antibiotics were not used by the recruited volunteers. Regarding daily fecal samples, three subjects had constipation and two subjects had frequent defecation. The fecal volume, shape/consistency, and color did not change over the week.

**Table 1:** Possible confounding variables regarding dietary consumption and defecation conditions for each subject (S1 to 10)^a,b^

	S1	S2	S3	S4	S5	S6	S7	S8	S9	S10	*P* value^i^
Seasonal stability											
Fermented milk (the beverages or yoghurt) (day/week)											
Season_1	0	1	6	7	0	0	0	0	0	6	0.022
Season_2	0	2	7	7	0	0	3	0	0	6	
Season_3	0	0	6	7	4	0	3	0	1	7	
Season_4	1	0	6	7	5	0	0	0	0	5	
Pro-/prebiotics (day/week)											
Season_1	0	0	0	0	0	0	0	0	0	0	NS
Season_2	0	0	0	0	0	0	0	0	0	0	
Season_3	0	0	0	0	0	0	0	0	0	0	
Season_4	0	0	0	0	0	0	0	0	0	0	
Antibiotics (day/week)											
Season_1	0	0	0	0	0	0	0	0	0	0	NS
Season_2	0	0	0	0	0	0	0	0	0	0	
Season_3	0	0	0	0	0	0	0	0	0	0	
Season_4	0	0	0	0	0	0	0	0	0	0	
Defecation											
Volume^c^	3.0	3.5	2.0	4.0	4.8	1.3	3.5	2.5	2.3	1.0	
	(2.3–3.0)	(3.0–4.0)	(1.6–3.5)	(3.5–4.5)	(3.8–4.9)	(1.0–1.9)	(2.3–4.0)	(2.1–2.9)	(2.0–2.9)	(0.6–1.0)	
Shape^d^	3–4	3–4	4–5	3–4	3	4	3–4	3–4	4	2–5	
Color^e^	C	D–E	C	A–B	C–D	B–E	D	B–D	B–C	D–E	
Daily stability											
Fermented milk (day/week)	1	0	6	7	5	0	0	0	0	6	
Pro-/prebiotics (day/week)	0	0	0	0	0	0	0	0	0	0	
Antibiotics (day/week)	0	0	0	0	0	0	0	0	0	0	
Defecation											
Days^f^	4	6	7	6	3	4	6	7	7	7	
Times^g^	4	7	7	6	3	4	6	7	12	16	
Interval^h^ (h)	36	23	23	24	45	46	29	24	13	4	
	(26–51)	(17–23)	(21–26)	(24–30)	(41–49)	(38–54)	(24–29)	(24–24)	(2–23)	(3–17)	
Volume^c^	2.3	4.0	2.5	2.5	3.0	1.5	4.0	3.0	1.0	1.5	
	(2.0–2.6)	(3.3–4.0)	(1.8–2.5)	(2.5–2.5)	(2.8–4.0)	(0.9–2.1)	(3.3–4.4)	(3.0–3.5)	(0.8–1.0)	(1.4–2.0)	
Shape^d^	3–4	2–5	3–4	4	3–4	4	4–6	1–4	3–4	1–3	
Color^e^	B–C	D–E	C	A	B–D	B–C	D–E	A–C	C–D	B–E	

*NS* not significant

^a^Seasonal and daily stabilities were defined as stabilities in the human gut microbiota for 1 year (i.e., 4 times representing each season) and 1 week (i.e., 7 continuous days), respectively

^b^Information on food intake and defecation and fecal samples were collected on defecation times in every quarter during 1 year, and at all defecation times during 1 week. We asked volunteers to provide information about such conditions 1 week and 1 day before their defecation, respectively. Thus, regarding daily stability, the data were summarized as the values for 1 week. The results on defecation represent the median (upper) and inter-quartile range (bottom) based on the data for each season during 1 year and 1 week (4 and at least 3 times)

^c^The stool volume was expressed as the number (e.g., 0.5, 1, and 2) of Japanese standard chicken egg size “S” (i.e., 40–52 g). The data represent the median (upper) and inter-quartile range (bottom) at different times (from 3 to 7 times)

^d^The numbers indicate the following: 1: watery, 2: muddy, 3: soft, 4: banana shape, 5: hard, or 6: very hard

^e^The letters represent the following: A: yellow, B: green ocher, C: brown, D: blackish brown, E: slightly blackish, or F: blackish

^f^Shape/consistency: the number of days with defecation in 1 week

^g^The number of defecation times in 1 week

^h^The data represent the median (upper) and inter-quartile range (bottom)

^i^Cochran’s *Q*-test

### Inter- and intra-individual variations for each intestinal human bacterium

**Fig. 1:**
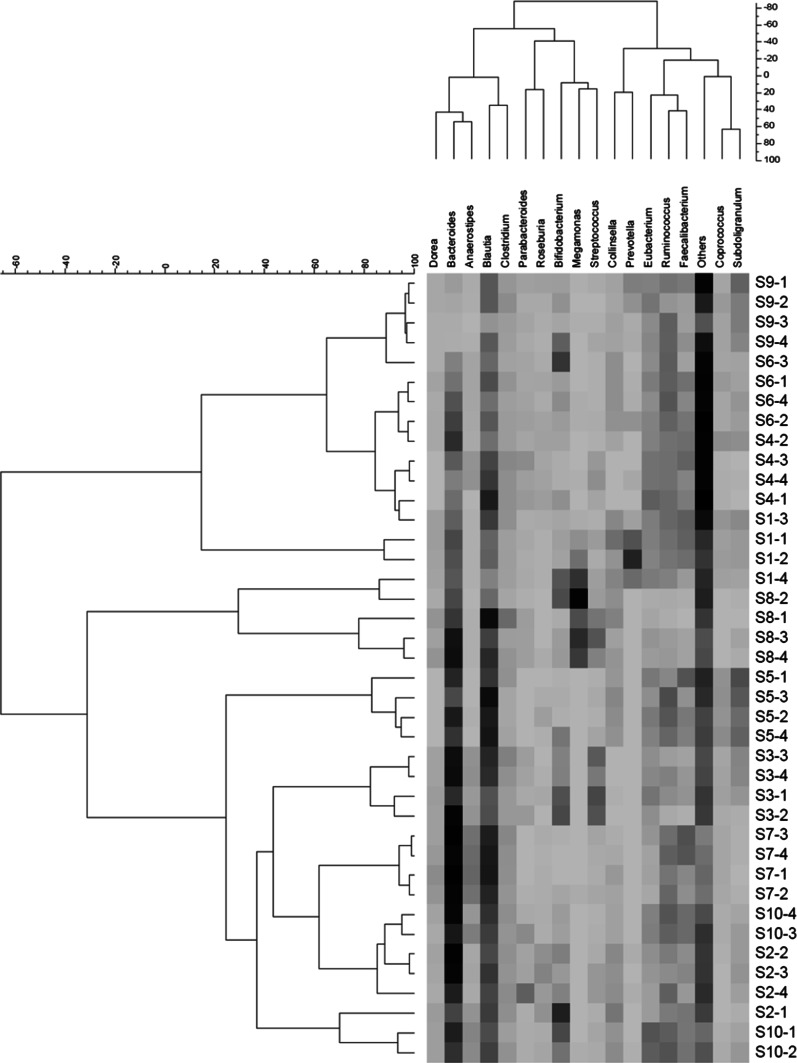
Double-hierarchical clustering of the 17 selected dominant genera and individual fecal samples collected for each season. As reference, the scale for the genus (representing >1.0 % of the total human gut microbiota) was attached on the *above panel* of the figure because it was not being outputted in the original figure. The genera and distances between them are depicted. On the *right* and *left*, samples are expressed by combined codes for the 10 subjects (S1–S10) and four seasons (1–4), and distances between the samples are depicted. Using the Ward method, each distance matrix between the genera or the samples is shown as Pearson’s correlation coefficient (*r*_p_)

**Fig. 2:**
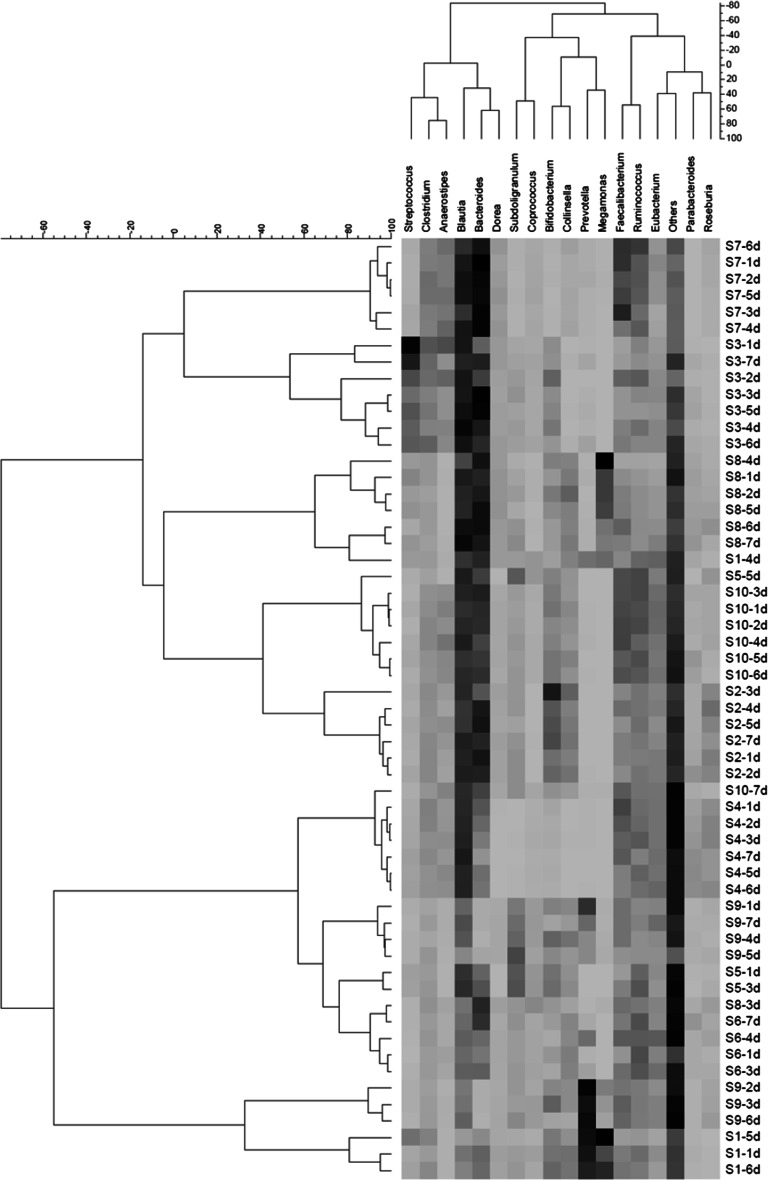
Double-hierarchical clustering of the 17 selected dominant genera and individual fecal samples collected for 7 continuous days. As reference, the scale for the genus (representing >1.0 % of the total human gut microbiota) was attached on the *above panel* of the figure because it was not being outputted in the original figure. The genera and distances between them are depicted. On the *right* and *left*, samples are expressed by combined codes for the 10 subjects (S1–S10) and 7 days (day 1–7), and distances between the samples are also depicted. Using the Ward method, each distance matrix between the genera or the samples is shown as Pearson’s correlation coefficient (*r*_p_)

We grouped each 16S rDNA genomic profile by using hierarchical clustering for the selected 17 genera representing >1.0 % of the total human gut microbiota (Figs. 1, 2). Regarding both seasonal and daily stabilities, no time trend was observed for each subject. Even if they were adjacent, consistent continuous orders (i.e., “season 1–4” or “day 1–7”) were not observed. Obviously, *r*_p_ were greater for daily than for seasonal stability. For both sets of samples, *Prevotella* and *Bacteroides* were classified in the two largest clusters, at the right and left, respectively (Figs. 1 and 2). Moreover, except *Bifidobacterium*, most *r*_s_ between *v*__four seasons_ and *v*__2d_ or *v*__3d_ % were similar or greater than those between *v*__four seasons_ and *v*__1d_ % (e.g., *r*_s_ = 0.38_0.89, 0.46_0.62, and 0.32_0.53, and 0.64_0.91, 0.78_0.95, and 0.79_0.95 for *v*__1d_ to *v*__3d_ % in *Bifidobacterium* and *Bacteroides*, respectively). Most of *r*_p_ were similar to the corresponding *r*_s_, but *r*_p_ in *Bifidobacterium*, *Prevotella*, *Megamonas*, and *Roseburia* were apparently greater (data not shown).

Means (SD) of each intestinal human bacterium in seasonal and daily fecal sample sets are presented in Tables 2 and 3. The following 39 genera (>0.1 % of the total human gut microbiota) were identified in the human gut microbiota: *Bifidobacterium*, *Collinsella*, *Eggerthella, Alistipes*, *Bacteroides*, *Barnesiella, Butyricimonas*, *Odoribacter*, *Parabacteroides*, *Prevotella*, *Acetivibrio*, *Anaerostipes*, *Bacillus*, *Blautia*, *Catenibacterium*, *Clostridium*, *Coprococcus*, *Dialister*, *Dorea*, *Eubacterium*, *Faecalibacterium*, *Lachnospira*, *Megamonas*, *Megasphaera*, *Mitsuokella*, *Oscillibacter*, *Phascolarctobacterium*, *Pseudoflavonifractor*, *Roseburia*, *Ruminococcus*, *Sporobacter*, *Streptococcus*, *Subdoligranulum*, *Veillonella*, *Brevundimonas*, *Mesorhizobium*, *Parasutterella*, *Sutterella*, and *Akkermansia* in the order listed in Tables 2 and 3. Significant inter-individual variations (i.e., differences between subjects) were found in 15 and 14 out of the selected 17 genera (>1.0 % of the total human gut microbiota) and in 9 out of the remaining 22 (0.1_1.0 % of them). Several genera such as *Prevotella* were not detected in some subjects, and the number of such genera was greater in the seasonal fecal sample set. Seasonal variation (differences between seasons), but not daily variation (differences between days), was found only for composition of *Dorea* (*P* < 0.05). Likewise, *P* values for “Subjects” and “Season” or “Day” based on Friedman test were almost the same (data not shown). Among the S2, S5, and S7 subjects, changes in *Bifidobacterium* composition might correspond with seasonal changes in fermented milk consumption (1.5 ± 2.0 and 5.8 ± 7.0 % for non-ingested and ingested seasons, respectively, Friedman test *P* = 0.08).

**Table 2:** Compositions (%) of 39 selected dominant genera in the human gut microbiota of the 10 subjects (S1–S10), based on seasonal stability

Phylum	Subject (S1–S10)					
Genus	S1	S2	S3	S4	S5	S6
Actinobacteria						
*Bifidobacterium*	4.0 ± 4.2^a^	8.6 ± 6.9	8.8 ± 3.6	1.6 ± 1.3	2.0 ± 2.8	6.2 ± 5.8
*Collinsella*	5.1 ± 1.6	4.7 ± 1.1	0.1 ± 0.1	0.4 ± 0.6	3.8 ± 0.7	3.7 ± 0.2
*Eggerthella*	0.1 ± 0.1	0.0 ± 0.0	0.2 ± 0.1	0.0 ± 0.0	0.0 ± 0.0	0.0 ± 0.0
Bacteroidetes						
*Alistipes*	1.2 ± 0.5	0.9 ± 0.7	0.5 ± 0.3	2.2 ± 0.5	1.2 ± 0.3	2.4 ± 0.8
*Bacteroides*	11.4 ± 1.3	24.0 ± 7.2	25.6 ± 4.6	10.4 ± 4.6	17.5 ± 3.3	9.7 ± 3.5
*Barnesiella*	0.4 ± 0.2	–	0.0 ± 0.0	0.8 ± 0.4	–	0.1 ± 0.0
*Butyricimonas*	0.1 ± 0.0	0.1 ± 0.1	0.0 ± 0.1	0.1 ± 0.2	0.1 ± 0.1	0.2 ± 0.0
*Odoribacter*	0.5 ± 0.5	0.0 ± 0.0	0.1 ± 0.1	0.2 ± 0.1	0.2 ± 0.0	0.3 ± 0.0
*Parabacteroides*	0.8 ± 0.2	3.3 ± 3.6	1.8 ± 0.7	2.5 ± 1.1	0.0 ± 0.0	1.5 ± 0.3
*Prevotella*	10.2 ± 6.5	0.3 ± 0.3	0.0 ± 0.0	0.3 ± 0.4	0.0 ± 0.0	1.0 ± 1.4
Firmicutes						
*Acetivibrio*	0.0 ± 0.0	–	0.0 ± 0.0	0.5 ± 0.3	0.0 ± 0.0	0.0 ± 0.0
*Anaerostipes*	0.2 ± 0.1	1.6 ± 0.9	3.2 ± 0.5	1.9 ± 1.5	0.1 ± 0.1	1.2 ± 0.1
*Bacillus*	0.0 ± 0.0	–	0.0 ± 0.0	0.1 ± 0.1	0.3 ± 0.3	0.0 ± 0.0
*Blautia*	9.7 ± 3.5	14.5 ± 1.3	15.2 ± 3.3	14.7 ± 5.1	22.2 ± 3.9	9.8 ± 1.9
*Catenibacterium*	0.0 ± 0.0	0.0 ± 0.1	0.0 ± 0.0	1.3 ± 1.6	–	0.0 ± 0.0
*Clostridium*	2.4 ± 0.7	3.6 ± 0.4	3.7 ± 1.1	3.6 ± 0.8	1.8 ± 0.9	2.3 ± 0.6
*Coprococcus*	2.3 ± 0.5	0.0 ± 0.0	1.3 ± 0.3	1.4 ± 1.6	3.6 ± 0.3	1.6 ± 0.7
*Dialister*	0.0 ± 0.0	0.0 ± 0.0	0.0 ± 0.0	0.0 ± 0.0	0.0 ± 0.0	0.8 ± 0.3
*Dorea*	1.8 ± 0.3	1.5 ± 0.5	1.9 ± 0.3	0.3 ± 0.4	0.0 ± 0.0	1.0 ± 0.3
*Eubacterium*	5.7 ± 0.4	2.9 ± 0.5	4.8 ± 1.3	7.4 ± 1.5	5.4 ± 1.1	4.8 ± 0.8
*Faecalibacterium*	7.4 ± 2.9	4.7 ± 1.5	2.0 ± 1.2	7.0 ± 1.5	6.7 ± 2.9	5.2 ± 2.2
*Lachnospira*	0.0 ± 0.0	1.6 ± 0.9	0.2 ± 0.2	1.0 ± 0.6	0.3 ± 0.2	0.6 ± 0.1
*Megamonas*	7.2 ± 6.1	0.0 ± 0.0	0.0 ± 0.0	0.1 ± 0.1	0.0 ± 0.0	0.2 ± 0.2
*Megasphaera*	1.7 ± 1.6	0.0 ± 0.0	0.0 ± 0.0	0.0 ± 0.0	–	0.7 ± 0.4
*Mitsuokella*	–	0.4 ± 0.7	–	–	–	0.6 ± 0.4
*Oscillibacter*	0.8 ± 0.4	0.1 ± 0.1	0.2 ± 0.1	1.3 ± 0.5	1.0 ± 0.2	0.6 ± 0.2
*Phascolarctobacterium*	1.0 ± 0.4	1.3 ± 0.5	0.1 ± 0.1	0.7 ± 0.4	1.4 ± 0.3	0.0 ± 0.0
*Pseudoflavonifractor*	0.2 ± 0.1	0.1 ± 0.1	0.1 ± 0.1	0.2 ± 0.1	0.4 ± 0.0	0.5 ± 0.1
*Roseburia*	0.4 ± 0.3	3.7 ± 0.9	0.3 ± 0.4	1.6 ± 0.4	0.7 ± 0.7	1.0 ± 0.4
*Ruminococcus*	7.1 ± 1.1	6.5 ± 1.9	3.2 ± 1.7	7.7 ± 0.4	9.2 ± 3.0	10.1 ± 0.7
*Sporobacter*	0.1 ± 0.1	0.0 ± 0.0	0.0 ± 0.0	0.2 ± 0.0	0.2 ± 0.1	0.4 ± 0.1
*Streptococcus*	1.3 ± 0.8	1.8 ± 1.1	10.4 ± 2.6	2.0 ± 1.3	0.7 ± 0.5	0.3 ± 0.2
*Subdoligranulum*	2.8 ± 1.1	2.1 ± 1.0	3.3 ± 1.8	1.0 ± 1.6	10.0 ± 1.8	2.2 ± 0.6
*Veillonella*	0.1 ± 0.1	0.2 ± 0.3	4.5 ± 1.8	0.1 ± 0.2	0.0 ± 0.0	0.0 ± 0.0
Proteobacteria						
*Brevundimonas*	0.1 ± 0.1	0.1 ± 0.1	0.0 ± 0.0	0.2 ± 0.4	0.0 ± 0.0	0.1 ± 0.1
*Mesorhizobium*	0.0 ± 0.0	0.0 ± 0.0	0.0 ± 0.0	0.1 ± 0.1	–	0.0 ± 0.0
*Parasutterella*	0.0 ± 0.0	1.5 ± 0.5	0.0 ± 0.0	0.1 ± 0.1	0.5 ± 0.1	0.4 ± 0.1
*Sutterella*	1.0 ± 0.2	0.0 ± 0.0	0.0 ± 0.0	0.1 ± 0.3	0.8 ± 0.2	0.0 ± 0.0
Verrucomicrobia						
*Akkermansia*	0.0 ± 0.1	0.0 ± 0.0	0.1 ± 0.2	1.1 ± 0.5	0.0 ± 0.0	0.3 ± 0.4
Others	12.9 ± 2.9	9.7 ± 0.5	8.2 ± 1.2	25.7 ± 3.8	9.8 ± 2.4	30.2 ± 4.0

Phylum	Subject (S1–S10)				*P* value^b^	
Genus	S7	S8	S9	S10	Subject	Season
Actinobacteria						
*Bifidobacterium*	0.3 ± 0.5	4.5 ± 4.7	4.1 ± 3.2	6.2 ± 4.5	0.001	0.740
*Collinsella*	1.5 ± 0.9	3.5 ± 1.3	1.5 ± 0.3	2.8 ± 1.1	0.001	0.077
*Eggerthella*	0.3 ± 0.1	0.1 ± 0.1	–^c^	0.1 ± 0.0	0.013	0.935
Bacteroidetes						
*Alistipes*	0.0 ± 0.0	0.0 ± 0.0	0.2 ± 0.1	0.7 ± 0.4	0.007	0.513
*Bacteroides*	34.3 ± 3.6	20.0 ± 5.7	1.2 ± 0.7	22.5 ± 4.7	<0.001	0.204
*Barnesiella*	–	–	0.0 ± 0.0	0.0 ± 0.0	0.152	0.226
*Butyricimonas*	0.2 ± 0.1	0.0 ± 0.0	0.3 ± 0.1	0.0 ± 0.0	0.006	0.378
*Odoribacter*	0.0 ± 0.0	0.0 ± 0.0	0.0 ± 0.1	0.2 ± 0.1	0.015	0.514
*Parabacteroides*	0.1 ± 0.0	1.6 ± 0.8	1.1 ± 0.4	1.6 ± 1.6	0.002	0.462
*Prevotella*	0.0 ± 0.0	0.0 ± 0.0	2.6 ± 2.0	0.1 ± 0.1	0.183	0.196
Firmicutes						
*Acetivibrio*	0.0 ± 0.0	0.0 ± 0.0	0.0 ± 0.0	–	0.274	0.400
*Anaerostipes*	7.9 ± 0.6	0.1 ± 0.1	0.3 ± 0.3	4.6 ± 1.1	0.026	0.715
*Bacillus*	0.3 ± 0.3	0.1 ± 0.2	0.2 ± 0.2	0.0 ± 0.1	0.019	0.443
*Blautia*	21.3 ± 1.4	17.2 ± 7.1	8.8 ± 3.2	14.7 ± 1.6	<0.001	0.382
*Catenibacterium*	0.0 ± 0.0	0.0 ± 0.0	4.9 ± 2.4	0.0 ± 0.0	0.237	0.541
*Clostridium*	3.7 ± 0.6	3.7 ± 2.7	2.9 ± 0.8	3.1 ± 0.6	<0.001	0.404
*Coprococcus*	1.2 ± 0.1	0.0 ± 0.0	1.7 ± 0.6	0.6 ± 0.1	0.003	0.547
*Dialister*	0.0 ± 0.0	1.3 ± 0.7	0.7 ± 0.4	0.0 ± 0.0	0.084	0.445
*Dorea*	3.0 ± 0.8	2.3 ± 1.0	0.8 ± 0.2	1.5 ± 0.4	0.001	0.016
*Eubacterium*	1.8 ± 0.8	1.8 ± 1.0	5.3 ± 1.0	8.2 ± 2.6	<0.001	0.129
*Faecalibacterium*	7.8 ± 3.7	1.0 ± 0.7	3.7 ± 1.7	7.5 ± 0.7	<0.001	0.504
*Lachnospira*	0.0 ± 0.0	1.0 ± 0.7	0.3 ± 0.2	0.2 ± 0.1	0.011	0.726
*Megamonas*	0.2 ± 0.3	20.3 ± 8.8	0.1 ± 0.1	0.1 ± 0.1	0.206	0.353
*Megasphaera*	0.0 ± 0.1	4.3 ± 3.9	0.0 ± 0.0	0.0 ± 0.0	0.156	0.387
*Mitsuokella*	–	0.0 ± 0.0	0.1 ± 0.1	–	0.119	0.469
*Oscillibacter*	0.0 ± 0.0	0.1 ± 0.0	1.3 ± 0.3	0.3 ± 0.2	0.006	0.599
*Phascolarctobacterium*	2.2 ± 0.4	0.0 ± 0.0	1.5 ± 0.3	1.1 ± 0.2	0.003	0.996
*Pseudoflavonifractor*	0.0 ± 0.0	0.2 ± 0.2	0.3 ± 0.1	0.1 ± 0.1	0.003	0.243
*Roseburia*	0.4 ± 0.2	0.2 ± 0.2	0.8 ± 0.7	0.8 ± 0.3	0.015	0.266
*Ruminococcus*	8.1 ± 1.0	1.6 ± 1.0	7.3 ± 2.7	10.5 ± 0.9	<0.001	0.203
*Sporobacter*	–	0.0 ± 0.0	1.0 ± 0.5	0.0 ± 0.0	0.096	0.253
*Streptococcus*	1.0 ± 0.3	6.4 ± 3.2	0.5 ± 0.4	0.7 ± 0.4	0.040	0.448
*Subdoligranulum*	0.1 ± 0.1	0.6 ± 0.5	6.6 ± 1.5	2.1 ± 0.7	0.011	0.672
*Veillonella*	0.1 ± 0.0	0.1 ± 0.1	0.0 ± 0.0	0.0 ± 0.0	0.270	0.379
Proteobacteria						
*Brevundimonas*	0.0 ± 0.0	0.0 ± 0.0	0.9 ± 1.2	0.1 ± 0.0	0.109	0.409
*Mesorhizobium*	0.0 ± 0.0	0.0 ± 0.0	0.1 ± 0.1	0.0 ± 0.0	0.173	0.683
*Parasutterella*	0.0 ± 0.0	0.0 ± 0.0	0.0 ± 0.0	0.0 ± 0.0	0.136	0.383
*Sutterella*	0.0 ± 0.0	0.0 ± 0.0	0.1 ± 0.1	1.4 ± 0.2	0.065	0.090
Verrucomicrobia						
*Akkermansia*	0.0 ± 0.0	0.0 ± 0.0	0.0 ± 0.0	1.1 ± 1.0	0.090	0.583
Others	4.0 ± 0.4	7.9 ± 3.5	38.9 ± 7.9	6.9 ± 1.7	0.003	0.215

^a^The individual mean ± SD was calculated using all values for 1 year

^b^According to one-way repeated measures ANOVA, the residual was defined as “Season.” The two variables, “Subject” and “Season,” correspond to “inter-” and “intra-” individual variations, respectively

^c^Not detected

**Table 3:** Compositions (%) of 39 selected dominant genera in the human gut microbiota for the 10 subjects (S1–S10), based on daily stability

Phylum	Subject (S1–S10)					
*Genus*	S1	S2	S3	S4	S5	S6
Actinobacteria						
*Bifidobacterium*	5.3 ± 2.2^a^	11.0 ± 4.4	4.4 ± 1.7	0.8 ± 0.2	5.9 ± 1.0	1.7 ± 0.8
*Collinsella*	5.1 ± 1.2	6.0 ± 1.1	0.1 ± 0.1	0.0 ± 0.0	3.2 ± 0.4	4.6 ± 0.4
*Eggerthella*	0.0 ± 0.0	0.1 ± 0.0	0.1 ± 0.1	0.1 ± 0.0	0.1 ± 0.1	0.0 ± 0.0
Bacteroidetes						
*Alistipes*	0.4 ± 0.4	0.7 ± 0.4	0.1 ± 0.1	1.9 ± 0.6	1.3 ± 0.2	2.7 ± 1.1
*Bacteroides*	8.9 ± 4.7	17.6 ± 3.9	18.5 ± 6.8	6.4 ± 2.0	10.1 ± 2.0	9.2 ± 3.5
*Barnesiella*	0.3 ± 0.3	0.0 ± 0.0	0.0 ± 0.0	0.7 ± 0.1	0.0 ± 0.0	0.1 ± 0.0
*Butyricimonas*	0.0 ± 0.0	0.0 ± 0.0	0.0 ± 0.0	–	0.0 ± 0.0	0.0 ± 0.0
*Odoribacter*	0.1 ± 0.2	–	0.0 ± 0.0	0.2 ± 0.1	0.2 ± 0.0	0.3 ± 0.0
*Parabacteroides*	0.5 ± 0.3	1.3 ± 0.8	0.7 ± 0.4	2.8 ± 0.7	0.0 ± 0.0	1.4 ± 0.9
*Prevotella*	17.4 ± 6.5	0.0 ± 0.0	0.5 ± 0.5	0.1 ± 0.0	0.0 ± 0.0	2.4 ± 2.7
Firmicutes						
*Acetivibrio*	0.0 ± 0.0	0.0 ± 0.0	0.0 ± 0.0	2.0 ± 0.3	0.0 ± 0.0	0.0 ± 0.0
*Anaerostipes*	0.3 ± 0.1	2.2 ± 0.7	5.2 ± 2.7	3.2 ± 0.5	0.1 ± 0.0	1.1 ± 0.3
*Bacillus*	0.1 ± 0.1	0.0 ± 0.0	0.0 ± 0.0	0.1 ± 0.0	0.2 ± 0.2	0.0 ± 0.0
*Blautia*	6.9 ± 4.5	16.3 ± 1.5	20.8 ± 2.3	17.3 ± 0.9	16.2 ± 1.6	7.9 ± 0.7
*Catenibacterium*	0.0 ± 0.0	0.0 ± 0.0	0.0 ± 0.0	0.4 ± 0.1	–	0.0 ± 0.0
*Clostridium*	3.3 ± 1.0	3.7 ± 0.3	6.9 ± 1.8	3.9 ± 0.6	2.1 ± 0.5	2.4 ± 0.3
*Coprococcus*	1.5 ± 0.8	0.0 ± 0.0	1.0 ± 0.2	0.5 ± 0.1	2.8 ± 0.1	1.3 ± 0.1
*Dialister*	–	0.0 ± 0.0	0.0 ± 0.0	0.0 ± 0.0	0.0 ± 0.0	1.3 ± 0.5
*Dorea*	1.6 ± 0.4	1.5 ± 0.2	2.0 ± 0.3	0.0 ± 0.0	0.0 ± 0.0	1.0 ± 0.1
*Eubacterium*	3.8 ± 2.5	4.7 ± 0.8	3.5 ± 0.7	6.5 ± 0.4	4.1 ± 0.8	6.3 ± 1.9
*Faecalibacterium*	4.5 ± 1.4	4.7 ± 1.1	4.0 ± 2.1	8.3 ± 2.7	6.1 ± 3.5	6.3 ± 2.3
*Lachnospira*	0.0 ± 0.0	2.8 ± 0.8	0.1 ± 0.1	1.1 ± 0.2	0.4 ± 0.1	0.9 ± 0.4
*Megamonas*	16.1 ± 8.1	0.0 ± 0.0	0.0 ± 0.0	0.0 ± 0.0	0.0 ± 0.0	0.0 ± 0.0
*Megasphaera*	3.2 ± 2.0	0.0 ± 0.0	0.0 ± 0.0	–	–	0.5 ± 0.1
*Mitsuokella*	0.0 ± 0.0	0.0 ± 0.0	0.0 ± 0.0	–	–	0.9 ± 0.6
*Oscillibacter*	0.4 ± 0.3	0.1 ± 0.1	0.1 ± 0.1	1.3 ± 0.4	2.4 ± 1.1	0.4 ± 0.1
*Phascolarctobacterium*	0.6 ± 0.3	0.9 ± 0.1	0.0 ± 0.0	0.6 ± 0.1	1.5 ± 0.3	0.0 ± 0.0
*Pseudoflavonifractor*	0.1 ± 0.1	0.1 ± 0.0	0.0 ± 0.0	0.2 ± 0.1	0.4 ± 0.1	0.6 ± 0.2
*Roseburia*	0.1 ± 0.1	4.7 ± 1.2	0.2 ± 0.2	3.7 ± 0.7	1.3 ± 1.1	1.1 ± 0.6
*Ruminococcus*	5.7 ± 1.9	5.9 ± 0.5	5.0 ± 2.0	7.2 ± 1.4	10.0 ± 1.4	10.4 ± 0.6
*Sporobacter*	0.0 ± 0.0	–	0.0 ± 0.0	0.2 ± 0.0	0.3 ± 0.1	0.2 ± 0.1
*Streptococcus*	2.7 ± 2.2	0.8 ± 0.3	12.9 ± 6.5	1.4 ± 0.4	1.2 ± 0.5	0.2 ± 0.1
*Subdoligranulum*	1.9 ± 0.7	2.9 ± 0.6	1.9 ± 0.6	0.1 ± 0.0	9.8 ± 0.5	2.7 ± 0.4
*Veillonella*	0.0 ± 0.0	0.0 ± 0.0	3.5 ± 1.5	0.0 ± 0.0	0.0 ± 0.0	0.0 ± 0.0
Proteobacteria						
*Brevundimonas*	0.0 ± 0.0	0.4 ± 0.2	0.7 ± 0.7	0.2 ± 0.1	0.0 ± 0.0	0.1 ± 0.0
*Mesorhizobium*	0.0 ± 0.0	0.1 ± 0.0	0.2 ± 0.2	0.0 ± 0.0	0.0 ± 0.0	0.0 ± 0.0
*Parasutterella*	0.0 ± 0.0	1.6 ± 0.3	0.0 ± 0.0	0.0 ± 0.0	0.4 ± 0.1	0.4 ± 0.1
*Sutterella*	1.1 ± 0.2	0.0 ± 0.0	0.0 ± 0.0	0.0 ± 0.0	0.6 ± 0.3	0.0 ± 0.0
Verrucomicrobia						
*Akkermansia*	0.0 ± 0.0	–	0.0 ± 0.0	0.4 ± 0.1	8.1 ± 6.0	0.0 ± 0.0
Others	8.1 ± 2.0	9.7 ± 0.5	7.3 ± 1.7	28.5 ± 2.2	11.2 ± 1.1	31.5 ± 5.4

Phylum	Subject (S1–S10)				*P* value^b^	
*Genus*	S7	S8	S9	S10	Subject	Day
Actinobacteria						
*Bifidobacterium*	0.1 ± 0.1	3.4 ± 1.2	4.6 ± 2.7	5.0 ± 1.0	0.002	0.397
*Collinsella*	1.1 ± 0.3	5.0 ± 1.9	3.4 ± 1.4	3.2 ± 0.7	0.003	0.749
*Eggerthella*	0.3 ± 0.1	0.3 ± 0.2	–^c^	0.1 ± 0.0	0.008	0.226
Bacteroidetes						
*Alistipes*	0.1 ± 0.1	0.4 ± 0.9	0.1 ± 0.1	0.8 ± 0.4	0.010	0.752
*Bacteroides*	27.9 ± 4.7	20.1 ± 2.5	0.6 ± 0.3	14.6 ± 1.9	<0.001	0.552
*Barnesiella*	0.0 ± 0.0	0.1 ± 0.1	0.0 ± 0.0	0.0 ± 0.0	0.125	0.786
*Butyricimonas*	0.4 ± 0.1	0.0 ± 0.0	0.0 ± 0.0	0.0 ± 0.0	0.343	0.435
*Odoribacter*	0.0 ± 0.0	0.1 ± 0.1	0.0 ± 0.0	0.2 ± 0.1	0.013	0.715
*Parabacteroides*	0.0 ± 0.0	1.7 ± 0.6	0.4 ± 0.2	1.2 ± 0.7	0.005	0.227
*Prevotella*	0.5 ± 0.2	0.1 ± 0.1	15.9 ± 10.9	0.1 ± 0.1	0.122	0.388
Firmicutes						
*Acetivibrio*	–	0.1 ± 0.2	0.0 ± 0.0	0.0 ± 0.0	0.305	0.254
*Anaerostipes*	6.4 ± 0.8	0.3 ± 0.4	0.4 ± 0.1	4.3 ± 0.8	0.010	0.552
*Bacillus*	0.0 ± 0.0	0.0 ± 0.0	0.1 ± 0.1	0.0 ± 0.0	0.040	0.612
*Blautia*	18.9 ± 2.8	16.7 ± 5.8	7.5 ± 2.1	16.1 ± 1.2	<0.001	0.353
*Catenibacterium*	0.0 ± 0.0	0.1 ± 0.1	2.4 ± 0.8	0.0 ± 0.0	0.265	0.447
*Clostridium*	5.7 ± 0.8	2.3 ± 0.4	2.0 ± 0.5	3.4 ± 0.5	<0.001	0.592
*Coprococcus*	1.0 ± 0.2	0.7 ± 1.4	2.0 ± 0.5	0.7 ± 0.1	0.001	0.312
*Dialister*	0.1 ± 0.0	1.0 ± 0.5	2.1 ± 0.4	0.0 ± 0.0	0.094	0.815
*Dorea*	2.5 ± 0.4	2.5 ± 0.4	1.0 ± 0.1	1.0 ± 0.1	0.001	0.616
*Eubacterium*	2.6 ± 1.0	3.0 ± 1.3	4.7 ± 1.2	6.5 ± 0.6	<0.001	0.625
*Faecalibacterium*	12.8 ± 3.6	5.1 ± 2.1	6.6 ± 1.6	10.8 ± 1.2	<0.001	0.683
*Lachnospira*	0.1 ± 0.0	0.7 ± 0.4	0.3 ± 0.3	0.3 ± 0.1	0.034	0.380
*Megamonas*	0.1 ± 0.1	11.2 ± 7.9	1.6 ± 1.6	0.0 ± 0.1	0.147	0.772
*Megasphaera*	0.0 ± 0.0	4.2 ± 3.4	0.0 ± 0.0	0.0 ± 0.0	0.142	0.583
*Mitsuokella*	–	0.0 ± 0.0	2.5 ± 2.4	0.0 ± 0.0	0.216	0.433
*Oscillibacter*	0.0 ± 0.0	0.3 ± 0.4	1.0 ± 0.7	0.4 ± 0.1	0.024	0.608
*Phascolarctobacterium*	1.8 ± 0.5	0.2 ± 0.3	0.4 ± 0.2	0.8 ± 0.2	0.006	0.221
*Pseudoflavonifractor*	0.0 ± 0.0	0.4 ± 0.2	0.2 ± 0.1	0.1 ± 0.0	0.006	0.761
*Roseburia*	0.7 ± 0.2	1.5 ± 0.8	1.1 ± 0.6	0.8 ± 0.4	0.011	0.173
*Ruminococcus*	9.8 ± 2.0	3.4 ± 1.4	4.7 ± 0.9	9.7 ± 1.8	<0.001	0.331
*Sporobacter*	0.0 ± 0.0	0.0 ± 0.0	0.6 ± 0.5	0.0 ± 0.0	0.073	0.613
*Streptococcus*	0.6 ± 0.2	2.2 ± 1.2	0.5 ± 0.1	0.6 ± 0.3	0.087	0.334
*Subdoligranulum*	0.2 ± 0.1	2.1 ± 1.1	5.8 ± 3.1	2.5 ± 0.6	0.010	0.618
*Veillonella*	0.0 ± 0.0	0.1 ± 0.0	0.0 ± 0.0	0.0 ± 0.0	0.319	0.454
Proteobacteria						
*Brevundimonas*	0.2 ± 0.2	1.0 ± 0.9	1.9 ± 2.6	4.7 ± 4.5	0.077	0.173
*Mesorhizobium*	0.1 ± 0.1	0.3 ± 0.3	0.2 ± 0.1	0.3 ± 0.2	0.006	0.230
*Parasutterella*	–	0.0 ± 0.0	0.0 ± 0.0	0.0 ± 0.0	0.164	0.456
*Sutterella*	0.0 ± 0.0	0.1 ± 0.2	0.2 ± 0.1	1.3 ± 0.4	0.073	0.127
Verrucomicrobia						
*Akkermansia*	0.0 ± 0.0	0.2 ± 0.6	0.1 ± 0.1	0.6 ± 0.6	0.268	0.433
Others	5.8 ± 1.0	9.2 ± 8.3	25.3 ± 10.9	9.4 ± 1.5	0.001	0.601

^a^The individual mean ± SD was calculated using all values for 1 week

^b^In one-way repeated measures ANOVA, the residual was defined as “Day.” The two variables “Subject” and “Day” correspond to “inter-” and “intra-” individual variations, respectively

^c^Not detected

### Relative contributions of intra-(*A*) and inter-individual variance (*B*) in each human intestinal bacterium

Relative contributions of intra-(*A*) and inter-individual variance (*B*) in each genus and the coefficients of within-person variance (CV_w_) and between-person variance (CV_b_) are presented in Tables 4 and 5. We also calculated the number of days of fecal sample collection required to estimate the true composition within 10 and 20 % of their true mean. In seasonal and daily fecal sample sets, A/B ratios for 26 and 35 out of the 39 selected genera were <1.0, except for *Bacillus*, *Megasphaera*, *Mitsuokella*, *Sporobacter,Brevundimonas,* and *Mesorhizobium*. In the two fecal sample sets, the mean values of each genus were similar, except for *Prevotella*, *Mitsuokella*, and *Brevundimonas* (1.5 vs. 3.5, 0.1 vs. 0.4, and 0.1 vs. 1.1 %, respectively). Compared with CV_b_, the values of CV_w_ were apparently greater for the seasonal fecal sample set than for the daily one, and most CV_b_ values were >100 % for both fecal sample sets. The former values in compositions of *Bifidobacterium* and *Dorea* were >2 times greater in the seasonal fecal sample set than in the daily one, and the latter values in those of 28 genera were >100 % in both or either fecal sample set. According to relatively greater latter values, it might be difficult to accurately estimate the true means, but significant or greater “differences between subjects” (i.e., inter-individual variance) were supported by the results presented in Tables 2, 3, 4, and 5.

**Table 4:** Relative contributions of intra- and inter-individual variance in 39 selected dominant genera, coefficient of within-person variance (CV_w_) and between-person variance (CV_b_), and the number of days (“Days”) of fecal sample collection required to estimate the true values within 10 and 20 % of their true mean, based on seasonal stability

Phylum	Percentage contributions of variance components^a^		*A*/*B*	Mean^b^	CV_w_	CV_b_	Days^c^	
*Genus*	Intra-individual (*A*)	Inter-individual (*B*)		(%)	(%)	(%)	10 %	20 %
Actinobacteria								
*Bifidobacterium*	90.8	9.2	9.8	4.6	94.6	107.5	344	86
*Collinsella*	28.5	71.5	0.4	2.7	52.6	70.7	106	27
*Eggerthella*	36.9	63.1	0.6	0.1	58.4	106.5	131	33
Bacteroidetes								
*Alistipes*	28.7	71.3	0.4	0.9	60.2	97.5	139	35
*Bacteroides*	22.2	77.8	0.3	17.7	27.9	57.2	30	7
*Barnesiella*	29.1	70.9	0.4	0.1	101.5	215.5	395	99
*Butyricimonas*	48.0	52.0	0.9	0.1	95.3	102.7	349	87
*Odoribacter*	65.1	34.9	1.9	0.2	88.4	137.5	300	75
*Parabacteroides*	84.5	15.5	5.4	1.4	66.7	116.3	171	43
*Prevotella*	43.0	57.0	0.8	1.5	95.1	257.4	347	87
Firmicutes								
*Acetivibrio*	46.6	53.4	0.9	0.1	112.6	293.6	487	122
*Anaerostipes*	9.8	90.2	0.1	2.1	42.9	117.3	71	18
*Bacillus*	91.3	8.7	10.5	0.1	125.7	182.5	607	152
*Blautia*	51.9	48.1	1.1	14.8	23.3	38.4	21	5
*Catenibacterium*	34.1	65.9	0.5	0.6	136.4	276.1	714	179
*Clostridium*	94.7	5.3	17.8	3.1	30.1	41.9	35	9
*Coprococcus*	34.3	65.7	0.5	1.4	42.7	86.4	70	18
*Dialister*	36.6	63.4	0.6	0.3	104.7	181.2	421	105
*Dorea*	31.4	68.6	0.5	1.4	51.5	71.1	102	25
*Eubacterium*	33.7	66.3	0.5	4.8	25.5	49.2	25	6
*Faecalibacterium*	57.4	42.6	1.3	5.3	41.6	59.5	67	17
*Lachnospira*	54.2	45.8	1.2	0.5	60.9	122.5	142	36
*Megamonas*	28.3	71.7	0.4	2.8	104.5	250.9	419	105
*Megasphaera*	65.3	34.7	1.9	0.7	105.5	276.3	427	107
*Mitsuokella*	79.9	20.1	4.0	0.1	118.3	288.6	538	134
*Oscillibacter*	28.3	71.7	0.4	0.6	52.6	94.3	106	27
*Phascolarctobacterium*	21.5	78.5	0.3	0.9	55.9	80.2	120	30
*Pseudoflavonifractor*	39.2	60.8	0.6	0.2	52.4	82.2	105	26
*Roseburia*	24.2	75.8	0.3	1.0	67.7	110.9	176	44
*Ruminococcus*	34.0	66.0	0.5	7.1	26.4	44.3	27	7
*Sporobacter*	34.1	65.9	0.5	0.2	88.5	184.2	301	75
*Streptococcus*	21.5	78.5	0.3	2.5	55.2	137.8	117	29
*Subdoligranulum*	18.1	81.9	0.2	3.1	53.5	101.3	110	28
*Veillonella*	18.9	81.1	0.2	0.5	89.3	274.3	306	77
Proteobacteria								
*Brevundimonas*	94.5	5.5	17.0	0.1	97.1	310.3	363	91
*Mesorhizobium*	92.7	7.3	12.8	0.0	110.6	261.4	470	117
*Parasutterella*	17.0	83.0	0.2	0.2	92.8	190.3	331	83
*Sutterella*	9.4	90.6	0.1	0.3	99.7	147.6	382	95
Verrucomicrobia								
*Akkermansia*	58.8	41.2	1.4	0.3	127.7	214.3	626	157
Others	10.8	89.2	0.1	15.4	19.6	76.2	15	4

^a^The inter-individual (*A*) variation represents variation between individual subjects, and intra-individual (*B*) variation represents variation in season and residual

^b^The mean of the composition (%) among the 10 subjects (all 40 samples)

^c^The number of days of fecal sample collection required to estimate the values within 10 and 20 % of their true mean with 95 % confidence

**Table 5:** Relative contributions of intra- and inter-individual variance in 39 selected dominant genera, coefficient of within-person variance (CV_w_) and between-person variance (CV_b_), and the number of days (“Days”) of fecal sample collection required to estimate the true values within 10 and 20 % of their true mean, based on daily stability

Phylum	Percentage contributions of variance components^a^		*A*/*B*	Mean^b^	CV_w_	CV_b_	Days^c^	
*Genus*	Intra-individual (*A*)	Inter-individual (*B*)		(%)	(%)	(%)	10 %	20 %
Actinobacteria								
*Bifidobacterium*	29.8	70.2	0.4	4.2	38.9	85.1	58	15
*Collinsella*	18.1	81.9	0.2	3.0	36.1	75.6	50	13
*Eggerthella*	41.6	58.4	0.7	0.1	40.0	103.8	61	15
Bacteroidetes								
*Alistipes*	27.2	72.8	0.4	0.7	74.7	125.8	214	54
*Bacteroides*	18.0	82.0	0.2	13.9	29.1	62.5	33	8
*Barnesiella*	15.6	84.4	0.2	0.1	142.3	208.5	778	195
*Butyricimonas*	11.0	89.0	0.1	0.0	115.3	290.1	511	128
*Odoribacter*	29.3	70.7	0.4	0.1	95.9	120.2	353	88
*Parabacteroides*	29.5	70.5	0.4	1.1	56.8	92.2	124	31
*Prevotella*	27.8	72.2	0.4	3.5	72.5	218.8	202	50
Firmicutes								
*Acetivibrio*	3.8	96.2	0.0	0.2	128.7	271.4	637	159
*Anaerostipes*	17.9	82.1	0.2	2.6	38.0	94.3	56	14
*Bacillus*	59.4	40.6	1.5	0.0	130.5	210.1	654	164
*Blautia*	24.2	75.8	0.3	14.9	19.5	37.0	15	4
*Catenibacterium*	12.2	87.8	0.1	0.3	154.2	241.4	913	228
*Clostridium*	20.2	79.8	0.3	3.7	18.8	48.3	14	3
*Coprococcus*	34.3	65.7	0.5	1.0	44.7	84.5	77	19
*Dialister*	11.7	88.3	0.1	0.5	105.5	164.4	428	107
*Dorea*	8.2	91.8	0.1	1.4	28.2	61.5	31	8
*Eubacterium*	42.0	58.0	0.7	4.6	27.6	40.2	29	7
*Faecalibacterium*	38.0	62.0	0.6	7.0	34.0	51.6	44	11
*Lachnospira*	13.8	86.2	0.2	0.7	58.8	132.5	133	33
*Megamonas*	27.4	72.6	0.4	2.7	101.3	229.7	394	99
*Megasphaera*	43.1	56.9	0.8	0.8	108.4	256.6	451	113
*Mitsuokella*	56.6	43.4	1.3	0.4	162.7	324.3	1017	254
*Oscillibacter*	23.3	76.7	0.3	0.6	59.3	131.8	135	34
*Phascolarctobacterium*	15.2	84.8	0.2	0.6	55.2	92.7	117	29
*Pseudoflavonifractor*	26.7	73.3	0.4	0.2	52.3	98.8	105	26
*Roseburia*	16.4	83.6	0.2	1.6	54.9	101.8	116	29
*Ruminococcus*	24.6	75.4	0.3	6.9	21.6	41.6	18	4
*Sporobacter*	47.2	52.8	0.9	0.1	98.3	213.1	371	93
*Streptococcus*	28.3	71.7	0.4	2.6	46.5	180.5	83	21
*Subdoligranulum*	15.0	85.0	0.2	2.7	32.6	98.3	41	10
*Veillonella*	16.9	83.1	0.2	0.5	74.1	275.4	211	53
Proteobacteria								
*Brevundimonas*	66.5	33.5	2.0	1.1	82.2	213.1	259	65
*Mesorhizobium*	70.7	29.3	2.4	0.2	76.6	119.1	226	56
*Parasutterella*	2.4	97.6	0.0	0.2	106.1	221.9	433	108
*Sutterella*	10.4	89.6	0.1	0.3	96.6	167.7	358	90
Verrucomicrobia								
*Akkermansia*	23.5	76.5	0.3	0.6	122.2	391.1	574	143
Others	21.7	78.3	0.3	14.3	25.5	73.0	25	6

^a^The inter-individual (*A*) variation represents variation between individual subjects, and intra-individual (*B*) variation represents day and residual variations

^b^Composition mean (%) among the 10 subjects (all 72 samples)

^c^The number of days of fecal sample collection required to estimate the intake values within 10 and 20 % of their true mean with 95 % confidence

## Discussion

In the seasonal and daily fecal sample sets, 39 genera representing >0.1 % of total bacteria were identified in the human gut microbiota and occupied approximately >85 % of the total human gut microbiota. In the two dendrograms for 17 genera that represent >1.0 % of the total human gut microbiota, no time trend of fecal sample collection was found and intra-individual variations were observed in seasonal and daily stabilities. However, compared with the intra-individual variation, a greater inter-individual variation was found to be statistically significant for almost all 39 selected dominant genera. This suggest that “differences between subjects” were detected based on their human gut bacterial community structure. Our data indicated that changes in the gut human microbiota composition in term of *Bifidobacterium*, but not *Lactobacillus*, corresponded to seasonal variations in fermented milk consumption.

Dietary assessment studies indicated that inter-individual variance was smaller than intra-individual variance in term of dietary intake of foods and nutrients (Ogawa et al. 1999; Tokudome et al. 2002). Similarly, among American-European individuals, A/B ratios have been reported to be smaller for dietary intake of macronutrients such as protein and carbohydrates, but not for that of minerals and vitamins. In our study, A/B ratios were also smaller for the 39 selected dominant genera, excluding few of them. Thus, similar to dietary assessments, bacterial community structure in the human gut allows the assessment of the intestinal microbiota composition for such dominant selected genera (and species; see supplemental Tables S1 to S4). However, the number of days of dietary information or fecal sample collection for both assessment of dietary foods/nutrient intake and bacterial community structure were not small to estimate the true values within 10 and 20 % of their true means. Even if *r*_s_ were adequately higher for some of the human intestinal bacteria, it might be difficult to precisely estimate point values for an individual subject, but not mean values in a group of subjects, because of CV_w_ greater values. This study was performed by using bacterial genus data to assess seasonal and daily stabilities of the human intestinal bacteria, but further investigation is needed to determine the relationships between bacterial species data, dietary habits, and health/diseases.

Large-scale projects such as the Human Microbiome Project and Meta-HIT have made substantial progress toward understanding the symbiotic relationships between the human gut microbiota and their hosts (The Human Microbiome Project Consortium 2012; Qin et al. 2012). According to cross-national cluster analyses, three enterotypes have been identified; i.e., *Bacteroides*, *Prevotella*, and *Ruminococcus* (Arumugam et al. 2011). *Bacteroides* and *Prevotella* compositions have been reported to be associated with host meals rich in animal proteins, several amino acids, and saturated fats as well as host diet featuring carbohydrates and monosaccharide (Wu et al. 2011; David et al. 2014). Moreover, higher consumption of fermented milk or administration of probiotics and prebiotics has been demonstrated to increase *Bifidobacterium* composition (Saito et al. 2002; Matsumoto et al. 2010; Petry et al. 2012). This study also found that changes in *Bifidobacterium* composition might correspond with seasonal changes in terms of fermented milk consumption, and this is related to greater values of intra-individual variation and greater A/B ratios for *Bifidobacterium*. However, further studies are needed to clarify the association between fermented milk intake and the proportions of *Bifidobacterium* and *Lactobacillus* in the microbiota for each season. In epidemiological studies, therefore, the composition of human intestinal bacteria has been shown to associate with change based on food ingestion or administration of supplements.

Individually, unique compositions of human intestinal bacterium were shown in 24 fecal samples derived from three collections of eight subjects without disease (Nam et al. 2011). Additionally, differences in some of human intestinal bacterium were found between healthy controls and patients with Crohn’s disease or ulcerative colitis (Gillevet et al. 2010). Through host inflammation, differences between human intestinal bacteria have been commonly shown to relate to the pathogenesis of cancer of the colon, stomach, breast, gallbladder, esophagus, and pancreas (Sheflin et al. 2014; Ohtani et al. 2014). In an animal study, the mechanism involving inflammation suggests that pathogenic species such as *Bacteroides fragilis* promote colorectal cancer and that *Fusobacterium* species are related to tumor progression (Tjalsma et al. 2012; McCoy et al. 2013).

This study has some limitations. The number of subjects in the study was small (*n* = 10), but 40 and 72 fecal samples were systematically collected to examine seasonal and daily stabilities of human intestinal bacteria, respectively. Bacterial community structure was assessed using the two sets of fecal samples from the same subjects. Inter- and intra-individual variations were examined only for a specific age group with equal gender ratio. However, taking into account the age and gender distribution, we appropriately recruited the subjects. In general, bacterial community structure was evaluated bacterial composition (%) because it was difficult to accurately evaluate “bacterial numbers per fecal dry or wet weight.” The data on specific species with >0.1 % of the total human gut microbiota are shown, whereas that on genera (and species) with <0.1 % were not shown, considering the measurement limitation for some of them. Regarding species classification, in this study, 83 species with >0.1 % of the total human gut microbiota were identified (i.e., 75 and 77, including 69 common, in seasonal and daily fecal sample sets, respectively), and a statistically significant greater inter-individual variation was observed for approximately 65 % of these (supplemental Tables S1 to S4). Eighteen identified dominant species within >1.0 % represented >45 % of the total human gut microbiota.

In conclusion, using the two sets of seasonal and daily fecal sample sets, we determined the following indices on human gut bacterial community structure: the intra- and inter-individual variance, the ratios, CV_w_ (%), CV_b_ (%), and the number of days of fecal sample collection required to estimate the compositions within 10 and 20 % of their true means. Compared with the intra-individual variance, a statistically significant greater inter-individual variation (“difference between persons”) was found for the 39 selected dominant genera. Our findings can be helpful to interpret human bacterial contribution to the role of human gut microbiota in nutritional metabolism, health promotion, and prevention/development of diseases in epidemiological and clinical studies.

## Electronic supplementary material

Supplementary material 1 (DOCX 45 kb)

Supplementary material 2 (DOCX 44 kb)

Supplementary material 3 (DOCX 38 kb)

Supplementary material 4 (DOCX 38 kb)

Supplementary material 5 (DOCX 50 kb)

Supplementary material 6 (DOCX 50 kb)
