# Supplementary material for: Inter- and intra-individual variations in seasonal and daily stabilities of the human gut microbiota in Japanese
Source: Arch Microbiol. 2015 Jun 12;197(7):919–34. doi: 10.1007/s00203-015-1125-0 (PMC4536265; doi:10.1007/s00203-015-1125-0)
Supplement: Supplementary file 1 — Supplementary material 1 (DOCX 45 kb) [file 203_2015_1125_MOESM1_ESM.docx]

**Table S1** Compositions (%) of 83 selected dominant species in the human gut microbiota of the 10 subjects (S1–S10), based on seasonal stability

| Phylum | Subject (S1–S10) | | | | | | | | | |  | *P* value^b^ | |
| --- | --- | --- | --- | --- | --- | --- | --- | --- | --- | --- | --- | --- | --- |
| *Species* | S1 | S2 | S3 | S4 | S5 | S6 | S7 | S8 | S9 | S10 |  | Subject | Season |
| Actinobacteria |  |  |  |  |  |  |  |  |  |  |  |  |  |
| *Bifidobacterium longum* | 0.6 ± 0.4^a^ | 3.5 ± 3.1 | 3.3 ± 1.8 | 1.1 ± 1.4 | 0.0 ± 0.0 | 0.1 ± 0.2 | 0.1 ± 0.1 | 1.5 ± 1.4 | 0.1 ± 0.0 | 1.8 ± 0.9 |  | 0.017 | 0.478 |
| *Collinsella aerofaciens* | 4.9 ± 1.6 | 4.7 ± 1.1 | 0.1 ± 0.1 | 0.4 ± 0.6 | 3.7 ± 0.7 | 3.6 ± 0.2 | 0.1 ± 0.1 | 3.4 ± 1.2 | 1.4 ± 0.2 | 2.7 ± 1.1 |  | 0.002 | 0.161 |
| *Eggerthella lenta* | − ^c^ | 0.0 ± 0.0 | 0.2 ± 0.1 | 0.0 ± 0.0 | 0.0 ± 0.0 | 0.0 ± 0.0 | 0.3 ± 0.1 | 0.1 ± 0.1 | − | 0.1 ± 0.0 |  | 0.029 | 0.907 |
| Bacteroidetes |  |  |  |  |  |  |  |  |  |  |  |  |  |
| *Alistipes onderdonkii* | 0.2 ± 0.2 | 0.0 ± 0.1 | 0.3 ± 0.2 | 0.2 ± 0.1 | − | 1.2 ± 0.4 | 0.0 ± 0.0 | 0.0 ± 0.0 | 0.0 ± 0.0 | 0.7 ± 0.4 |  | 0.055 | 0.089 |
| *Alistipes putredinis* | 0.5 ± 0.2 | 0.7 ± 0.4 | 0.1 ± 0.1 | 1.1 ± 0.2 | 0.5 ± 0.2 | 0.1 ± 0.0 | 0.0 ± 0.0 | − | 0.1 ± 0.1 | 0.0 ± 0.0 |  | 0.028 | 0.589 |
| *Alistipes shahii* | 0.2 ± 0.2 | 0.0 ± 0.0 | 0.0 ± 0.0 | 0.6 ± 0.2 | 0.5 ± 0.2 | 0.6 ± 0.2 | 0.0 ± 0.0 | − | 0.0 ± 0.0 | 0.0 ± 0.0 |  | 0.039 | 0.206 |
| *Bacteroides clarus* | 0.0 ± 0.0 | 0.8 ± 0.8 | 0.3 ± 0.2 | − | − | 0.0 ± 0.0 | − | − | − | − |  | 0.221 | 0.476 |
| *Bacteroides coprocola* | 3.8 ± 1.7 | 0.0 ± 0.1 | 0.4 ± 0.6 | 1.3 ± 2.2 | 0.0 ± 0.0 | 0.0 ± 0.0 | 0.0 ± 0.0 | 0.0 ± 0.0 | 0.1 ± 0.1 | 0.0 ± 0.0 |  | 0.176 | 0.293 |
| *Bacteroides dorei* | 0.2 ± 0.2 | 3.1 ± 0.6 | 0.4 ± 0.2 | 0.6 ± 0.9 | 11.6 ± 2.1 | 2.0 ± 0.6 | 5.2 ± 0.4 | 3.6 ± 2.0 | 0.1 ± 0.1 | 0.0 ± 0.0 |  | 0.044 | 0.488 |
| *Bacteroides eggerthii* | 0.3 ± 0.5 | 1.0 ± 0.5 | − | − | − | 0.2 ± 0.1 | − | − | − | − |  | 0.175 | 0.419 |
| *Bacteroides faecichinchillae* | 0.0 ± 0.0 | 0.6 ± 0.5 | 0.0 ± 0.0 | 0.1 ± 0.1 | 0.6 ± 0.5 | 0.5 ± 0.1 | 1.3 ± 0.4 | 0.0 ± 0.0 | 0.0 ± 0.0 | 0.0 ± 0.0 |  | 0.046 | 0.216 |
| *Bacteroides faecis* | 0.1 ± 0.0 | 0.0 ± 0.0 | − | 0.0 ± 0.0 | 0.0 ± 0.0 | 0.2 ± 0.1 | 1.7 ± 0.4 | 0.5 ± 0.6 | 0.0 ± 0.0 | 0.3 ± 0.2 |  | 0.136 | 0.346 |
| *Bacteroides finegoldii* | 0.1 ± 0.0 | 0.0 ± 0.0 | 0.0 ± 0.1 | 0.2 ± 0.2 | 0.0 ± 0.0 | 0.1 ± 0.0 | 0.1 ± 0.0 | 0.0 ± 0.0 | − | 1.5 ± 0.5 |  | 0.204 | 0.672 |
| *Bacteroides fragilis* | − | 0.1 ± 0.0 | 0.3 ± 0.2 | 0.2 ± 0.2 | 0.0 ± 0.0 | 0.0 ± 0.0 | 0.0 ± 0.0 | 0.7 ± 0.9 | 0.0 ± 0.1 | 0.1 ± 0.1 |  | 0.068 | 0.250 |
| *Bacteroides massiliensis* | 1.8 ± 0.4 | 3.3 ± 1.7 | 0.0 ± 0.0 | 0.0 ± 0.0 | − | 0.0 ± 0.0 | 0.0 ± 0.0 | − | − | 0.0 ± 0.0 |  | 0.186 | 0.296 |
| *Bacteroides ovatus* | 0.1 ± 0.0 | 0.0 ± 0.0 | 1.3 ± 0.7 | 0.4 ± 0.2 | 1.7 ± 0.6 | 0.3 ± 0.2 | 7.3 ± 1.8 | 0.0 ± 0.0 | 0.0 ± 0.0 | 0.6 ± 0.2 |  | 0.123 | 0.345 |
| *Bacteroides plebeius* | 0.1 ± 0.0 | 0.0 ± 0.0 | 7.6 ± 3.9 | 0.0 ± 0.0 | 0.0 ± 0.0 | 3.0 ± 1.9 | 0.2 ± 0.3 | 12.4 ± 4.8 | 0.0 ± 0.0 | 0.1 ± 0.1 |  | 0.118 | 0.307 |
| *Bacteroides stercoris* | 0.5 ± 0.1 | 0.0 ± 0.0 | 0.0 ± 0.0 | 0.2 ± 0.2 | 1.6 ± 0.2 | 0.1 ± 0.1 | 0.0 ± 0.0 | 0.9 ± 1.1 | 0.1 ± 0.1 | 0.0 ± 0.0 |  | 0.076 | 0.386 |
| *Bacteroides uniformis* | 1.3 ± 0.5 | 3.2 ± 1.0 | 0.9 ± 0.2 | 4.5 ± 1.9 | 1.0 ± 0.6 | 1.4 ± 0.8 | 7.6 ± 1.6 | 0.8 ± 0.6 | 0.2 ± 0.1 | 0.0 ± 0.0 |  | 0.022 | 0.888 |
| *Bacteroides vulgatus* | 2.7 ± 1.1 | 8.8 ± 2.4 | 12.8 ± 3.5 | 1.2 ± 1.9 | 0.1 ± 0.2 | 0.5 ± 0.1 | 9.5 ± 2.5 | 0.1 ± 0.2 | 0.3 ± 0.2 | 17.4 ± 3.9 |  | 0.026 | 0.468 |
| *Bacteroides xylanisolvens* | 0.0 ± 0.0 | 1.7 ± 1.2 | 0.6 ± 0.3 | 0.8 ± 0.6 | 0.1 ± 0.2 | 0.1 ± 0.1 | 0.2 ± 0.1 | 0.5 ± 0.4 | 0.0 ± 0.0 | 2.0 ± 0.4 |  | 0.024 | 0.556 |
| *Barnesiella intestinihominis* | 0.4 ± 0.2 | − | 0.0 ± 0.0 | 0.8 ± 0.4 | − | 0.1 ± 0.0 | − | − | 0.0 ± 0.0 | 0.0 ± 0.0 |  | 0.152 | 0.226 |
| *Odoribacter splanchnicus* | 0.1 ± 0.1 | 0.0 ± 0.0 | 0.1 ± 0.1 | 0.1 ± 0.1 | 0.2 ± 0.0 | 0.3 ± 0.1 | 0.0 ± 0.0 | 0.0 ± 0.0 | 0.0 ± 0.1 | 0.2 ± 0.1 |  | 0.009 | 0.826 |
| *Parabacteroides distasonis* | 0.2 ± 0.1 | 3.2 ± 3.6 | 1.7 ± 0.7 | 1.8 ± 0.8 | 0.0 ± 0.0 | 0.5 ± 0.2 | 0.0 ± 0.0 | 0.7 ± 0.4 | 0.7 ± 0.2 | 0.0 ± 0.0 |  | 0.027 | 0.420 |
| *Parabacteroides johnsonii* | − | − | − | − | − | − | 0.0 ± 0.0 | 0.0 ± 0.0 | 0.0 ± 0.0 | 1.5 ± 1.6 |  | 0.311 | 0.307 |
| *Parabacteroides merdae* | 0.5 ± 0.2 | 0.0 ± 0.0 | 0.0 ± 0.0 | − | 0.0 ± 0.0 | 0.7 ± 0.2 | 0.0 ± 0.0 | 0.9 ± 0.5 | 0.2 ± 0.2 | 0.0 ± 0.0 |  | 0.060 | 0.166 |
| *Prevotella copri* | 6.2 ± 3.4 | 0.1 ± 0.1 | 0.0 ± 0.0 | 0.2 ± 0.3 | 0.0 ± 0.0 | 0.9 ± 1.3 | 0.0 ± 0.0 | 0.0 ± 0.0 | 1.8 ± 1.7 | 0.0 ± 0.0 |  | 0.164 | 0.184 |
| *Prevotella stercorea* | 0.0 ± 0.0 | 0.0 ± 0.0 | − | − | − | 0.0 ± 0.0 | 0.0 ± 0.0 | − | 0.3 ± 0.2 | 0.0 ± 0.0 |  | 0.255 | 0.134 |

to be continued

**Table S1** continued

| Phylum | Subject (S1–S10) | | | | | | | | | |  | *P* value | |
| --- | --- | --- | --- | --- | --- | --- | --- | --- | --- | --- | --- | --- | --- |
| *Species* | S1 | S2 | S3 | S4 | S5 | S6 | S7 | S8 | S9 | S10 |  | Subject | Season |
| Firmicutes |  |  |  |  |  |  |  |  |  |  |  |  |  |
| *Blautia faecis* | 0.6 ± 0.3 | 0.3 ± 0.1 | 0.4 ± 0.2 | 1.7 ± 0.6 | 1.9 ± 1.6 | 0.5 ± 0.1 | 0.5 ± 0.2 | 0.2 ± 0.2 | 1.3 ± 0.9 | 0.7 ± 0.3 |  | 0.002 | 0.543 |
| *Blautia glucerasea* | 0.0 ± 0.0 | 0.3 ± 0.2 | 0.2 ± 0.1 | 0.1 ± 0.1 | 0.0 ± 0.0 | 0.0 ± 0.0 | 0.1 ± 0.0 | 0.8 ± 0.8 | 0.0 ± 0.0 | 0.0 ± 0.0 |  | 0.091 | 0.397 |
| *Blautia luti* | 2.9 ± 1.0 | 2.4 ± 0.3 | 7.6 ± 2.3 | 2.3 ± 0.6 | 14.3 ± 3.1 | 4.0 ± 1.0 | 0.2 ± 0.1 | 1.6 ± 1.0 | 1.0 ± 0.3 | 5.4 ± 1.0 |  | 0.011 | 0.217 |
| *Blautia stercoris* | 0.6 ± 0.4 | 1.1 ± 0.6 | 0.0 ± 0.0 | 0.0 ± 0.0 | 0.0 ± 0.0 | 0.0 ± 0.0 | 0.6 ± 0.2 | − | 0.1 ± 0.0 | 0.0 ± 0.0 |  | 0.078 | 0.362 |
| *Blautia wexlerae* | 3.1 ± 0.9 | 7.2 ± 0.6 | 3.9 ± 1.0 | 8.2 ± 3.5 | 4.6 ± 2.8 | 3.1 ± 1.2 | 15.9 ± 0.7 | 6.6 ± 1.3 | 4.3 ± 1.9 | 7.2 ± 1.5 |  | <0.001 | 0.465 |
| *Catenibacterium mitsuokai* | − | 0.0 ± 0.0 | 0.0 ± 0.0 | 0.5 ± 0.6 | − | 0.0 ± 0.0 | − | 0.0 ± 0.0 | 1.4 ± 0.8 | 0.0 ± 0.0 |  | 0.223 | 0.813 |
| *Clostridium bartlettii* | 0.1 ± 0.1 | 0.3 ± 0.2 | 0.1 ± 0.1 | 0.1 ± 0.1 | 0.2 ± 0.3 | 0.1 ± 0.1 | 0.3 ± 0.3 | 0.6 ± 0.7 | 0.0 ± 0.1 | 0.1 ± 0.0 |  | 0.007 | 0.389 |
| *Clostridium celerecrescens* | 0.0 ± 0.0 | 0.0 ± 0.0 | − | 0.0 ± 0.0 | 0.0 ± 0.0 | 0.0 ± 0.0 | 0.0 ± 0.0 | 2.0 ± 1.8 | 0.0 ± 0.0 | 0.0 ± 0.0 |  | 0.331 | 0.391 |
| *Clostridium disporicum* | 0.0 ± 0.0 | 0.2 ± 0.2 | 0.0 ± 0.0 | 0.1 ± 0.1 | 0.0 ± 0.0 | 0.1 ± 0.1 | − | 0.1 ± 0.2 | 0.0 ± 0.0 | 0.1 ± 0.1 |  | 0.010 | 0.942 |
| *Clostridium leptum* | 0.3 ± 0.4 | 0.4 ± 0.4 | 0.1 ± 0.1 | 0.0 ± 0.0 | 0.1 ± 0.1 | 0.1 ± 0.1 | 0.1 ± 0.1 | 0.0 ± 0.0 | 0.1 ± 0.1 | 0.2 ± 0.2 |  | 0.006 | 0.639 |
| *Clostridium lituseburense* | 0.3 ± 0.2 | 0.2 ± 0.1 | 0.0 ± 0.0 | 0.3 ± 0.2 | 0.2 ± 0.1 | 0.4 ± 0.4 | 0.0 ± 0.0 | 0.7 ± 0.7 | 0.1 ± 0.1 | 0.2 ± 0.1 |  | 0.008 | 0.625 |
| *Clostridium xylanolyticum* | 0.0 ± 0.0 | 0.0 ± 0.0 | 0.0 ± 0.0 | 0.8 ± 0.8 | 0.0 ± 0.0 | 0.3 ± 0.3 | 0.1 ± 0.0 | 0.0 ± 0.0 | 0.1 ± 0.1 | 0.0 ± 0.0 |  | 0.150 | 0.498 |
| *Coprococcus catus* | 0.5 ± 0.2 | 0.0 ± 0.0 | 0.1 ± 0.1 | 0.5 ± 0.2 | 0.0 ± 0.0 | 0.6 ± 0.2 | 0.0 ± 0.0 | 0.0 ± 0.0 | 0.4 ± 0.1 | 0.5 ± 0.1 |  | 0.014 | 0.329 |
| *Coprococcus comes* | 0.4 ± 0.1 | 0.0 ± 0.0 | 1.0 ± 0.3 | 0.1 ± 0.2 | 2.9 ± 0.1 | 0.6 ± 0.1 | 1.1 ± 0.1 | 0.0 ± 0.0 | 1.0 ± 0.4 | 0.1 ± 0.1 |  | 0.027 | 0.424 |
| *Coprococcus eutactus* | 1.2 ± 0.5 | 0.0 ± 0.0 | 0.2 ± 0.3 | 0.7 ± 1.1 | 0.5 ± 0.3 | 0.3 ± 0.5 | 0.0 ± 0.0 | 0.0 ± 0.0 | 0.2 ± 0.2 | 0.0 ± 0.0 |  | 0.035 | 0.775 |
| *Dialister succinatiphilus* | − | − | − | − | − | 0.0 ± 0.0 | 0.0 ± 0.0 | 1.3 ± 0.7 | 0.0 ± 0.0 | 0.0 ± 0.0 |  | 0.340 | 0.408 |
| *Dorea formicigenerans* | 0.4 ± 0.1 | 0.1 ± 0.2 | 0.3 ± 0.1 | 0.1 ± 0.1 | 0.0 ± 0.0 | 0.3 ± 0.2 | 0.0 ± 0.0 | 0.5 ± 0.3 | 0.2 ± 0.0 | 0.3 ± 0.1 |  | 0.004 | 0.030 |
| *Dorea longicatena* | 1.4 ± 0.2 | 1.3 ± 0.3 | 1.6 ± 0.2 | 0.2 ± 0.3 | 0.0 ± 0.0 | 0.6 ± 0.1 | 3.0 ± 0.8 | 1.7 ± 0.7 | 0.6 ± 0.2 | 1.2 ± 0.4 |  | 0.002 | 0.033 |
| *Eubacterium cprostanoligenes* | 0.0 ± 0.0 | 0.0 ± 0.0 | 0.1 ± 0.1 | 0.4 ± 0.1 | − | 0.0 ± 0.0 | 0.0 ± 0.0 | 0.0 ± 0.0 | 0.4 ± 0.2 | 0.0 ± 0.0 |  | 0.145 | 0.066 |
| *Eubacterium desmolans* | 0.0 ± 0.0 | 0.3 ± 0.1 | 0.1 ± 0.1 | 0.1 ± 0.1 | 0.0 ± 0.0 | 0.0 ± 0.0 | 0.0 ± 0.0 | 0.0 ± 0.0 | 0.0 ± 0.0 | 0.0 ± 0.0 |  | 0.196 | 0.301 |
| *Eubacterium eligens* | 0.0 ± 0.0 | 0.0 ± 0.0 | 0.0 ± 0.0 | 0.8 ± 0.6 | − | 0.1 ± 0.2 | 1.0 ± 0.8 | 0.0 ± 0.0 | 0.1 ± 0.1 | 0.0 ± 0.0 |  | 0.117 | 0.439 |
| *Eubacterium hadrum* | 0.2 ± 0.1 | 1.6 ± 0.9 | 3.2 ± 0.5 | 1.8 ± 1.4 | 0.0 ± 0.0 | 1.1 ± 0.1 | 7.7 ± 0.7 | 0.0 ± 0.0 | 0.3 ± 0.3 | 4.5 ± 1.1 |  | 0.028 | 0.779 |
| *Eubacterium hallii* | 1.1 ± 0.4 | 1.8 ± 0.5 | 1.2 ± 0.2 | 1.7 ± 0.5 | 2.7 ± 0.5 | 1.5 ± 0.3 | 0.0 ± 0.0 | 1.5 ± 0.7 | 2.0 ± 0.9 | 2.0 ± 0.1 |  | <0.001 | 0.941 |
| *Eubacterium ramulus* | 0.3 ± 0.1 | 0.0 ± 0.0 | 0.2 ± 0.1 | 0.0 ± 0.0 | 0.5 ± 0.1 | 0.1 ± 0.0 | 0.0 ± 0.0 | 0.0 ± 0.0 | 0.1 ± 0.0 | 0.2 ± 0.1 |  | 0.016 | 0.383 |
| *Eubacterium rectale* | 2.2 ± 0.7 | 0.0 ± 0.0 | 1.6 ± 1.4 | 0.1 ± 0.1 | 0.0 ± 0.0 | 0.1 ± 0.1 | 0.0 ± 0.0 | 0.0 ± 0.0 | 0.2 ± 0.2 | 4.1 ± 2.2 |  | 0.090 | 0.235 |
| *Eubacterium ruminantium* | 0.7 ± 0.4 | 0.0 ± 0.0 | 0.0 ± 0.0 | 0.5 ± 0.6 | − | 1.3 ± 0.1 | 0.0 ± 0.0 | 0.0 ± 0.0 | 0.1 ± 0.1 | − |  | 0.102 | 0.188 |
| *Eubacterium siraeum* | 0.0 ± 0.0 | 0.0 ± 0.0 | − | 2.0 ± 1.1 | 0.5 ± 0.5 | 0.4 ± 0.2 | − | 0.0 ± 0.0 | 1.9 ± 0.6 | 0.0 ± 0.0 |  | 0.091 | 0.592 |
| *Eubacterium ventriosum* | 0.1 ± 0.0 | 0.2 ± 0.2 | 0.3 ± 0.2 | 0.4 ± 0.3 | 0.4 ± 0.1 | 0.3 ± 0.2 | 0.5 ± 0.3 | 0.0 ± 0.0 | 0.0 ± 0.0 | 0.5 ± 0.4 |  | 0.002 | 0.061 |
| *Faecalibacterium prausnitzii* | 7.3 ± 2.9 | 4.6 ± 1.5 | 1.9 ± 1.2 | 6.8 ± 1.3 | 6.5 ± 2.8 | 5.1 ± 2.2 | 7.7 ± 3.6 | 0.9 ± 0.7 | 3.6 ± 1.8 | 7.3 ± 0.7 |  | <0.001 | 0.491 |
| *Lachnospira pectinoschiza* | 0.0 ± 0.0 | 1.2 ± 0.8 | − | 0.1 ± 0.1 | 0.0 ± 0.0 | 0.0 ± 0.0 | − | 0.1 ± 0.1 | 0.0 ± 0.0 | 0.0 ± 0.0 |  | 0.257 | 0.318 |
| *Megamonas funiformis* | 7.0 ± 6.0 | 0.0 ± 0.0 | 0.0 ± 0.0 | 0.1 ± 0.1 | 0.0 ± 0.0 | 0.2 ± 0.2 | 0.1 ± 0.2 | 19.5 ± 8.5 | 0.1 ± 0.1 | 0.1 ± 0.1 |  | 0.206 | 0.355 |
| *Megamonas rupellensis* | 0.0 ± 0.0 | − | − | 0.0 ± 0.0 | − | 0.0 ± 0.0 | 0.0 ± 0.0 | 0.2 ± 0.2 | 0.0 ± 0.0 | − |  | 0.343 | 0.408 |

to be continued

**Table S1** continue

| Phylum | Subject (S1–S10) | | | | | | | | | |  | *P* value | |
| --- | --- | --- | --- | --- | --- | --- | --- | --- | --- | --- | --- | --- | --- |
| *Species* | S1 | S2 | S3 | S4 | S5 | S6 | S7 | S8 | S9 | S10 |  | Subject | Season |
| *Megasphaera elsdenii* | 1.5 ± 1.4 | 0.0 ± 0.0 | − | 0.0 ± 0.0 | − | 0.7 ± 0.4 | 0.0 ± 0.1 | 3.9 ± 3.5 | 0.0 ± 0.0 | 0.0 ± 0.0 |  | 0.153 | 0.394 |
| *Mitsuokella multacida* | − | 0.4 ± 0.7 | − | − | − | 0.5 ± 0.4 | − | − | 0.1 ± 0.1 | − |  | 0.118 | 0.402 |
| *Phascolarctobacterium faecium* | 0.0 ± 0.0 | 0.0 ± 0.0 | − | 0.4 ± 0.2 | 0.0 ± 0.0 | 0.0 ± 0.0 | 2.1 ± 0.4 | 0.0 ± 0.0 | 0.0 ± 0.1 | 1.0 ± 0.2 |  | 0.143 | 0.273 |
| *Phascolarctobacterium succinatutens* | 0.0 ± 0.0 | 1.2 ± 0.5 | 0.1 ± 0.1 | 0.3 ± 0.5 | 0.0 ± 0.0 | 0.0 ± 0.0 | 0.0 ± 0.0 | 0.0 ± 0.0 | 1.4 ± 0.4 | 0.0 ± 0.0 |  | 0.118 | 0.535 |
| *Roseburia faecis* | 0.1 ± 0.0 | 1.3 ± 1.0 | 0.2 ± 0.3 | 1.1 ± 0.5 | 0.0 ± 0.0 | 0.5 ± 0.2 | 0.0 ± 0.0 | 0.1 ± 0.2 | 0.4 ± 0.6 | 0.4 ± 0.2 |  | 0.019 | 0.051 |
| *Roseburia intestinalis* | 0.0 ± 0.0 | 1.8 ± 1.5 | 0.0 ± 0.0 | 0.2 ± 0.2 | 0.6 ± 0.7 | 0.0 ± 0.0 | 0.0 ± 0.0 | − | 0.2 ± 0.1 | 0.1 ± 0.3 |  | 0.121 | 0.138 |
| *Roseburia inulinivorans* | 0.1 ± 0.1 | 0.5 ± 0.3 | 0.0 ± 0.1 | 0.0 ± 0.0 | − | 0.3 ± 0.1 | 0.4 ± 0.2 | 0.0 ± 0.0 | 0.1 ± 0.1 | 0.2 ± 0.2 |  | 0.015 | 0.840 |
| *Ruminococcus bromii* | 0.6 ± 0.2 | 0.0 ± 0.0 | 0.3 ± 0.5 | 4.8 ± 1.5 | 4.5 ± 0.9 | 3.9 ± 0.9 | 0.0 ± 0.0 | 0.0 ± 0.0 | 3.2 ± 2.0 | 1.7 ± 2.1 |  | 0.015 | 0.723 |
| *Ruminococcus callidus* | 1.0 ± 0.2 | − | 0.0 ± 0.0 | 0.7 ± 0.9 | 1.0 ± 0.2 | 0.2 ± 0.1 | 0.0 ± 0.0 | 0.0 ± 0.0 | 0.0 ± 0.0 | 0.6 ± 0.7 |  | 0.026 | 0.785 |
| *Ruminococcus faecis* | 1.5 ± 0.4 | 0.0 ± 0.0 | 0.1 ± 0.1 | 0.3 ± 0.5 | 0.0 ± 0.0 | 3.5 ± 0.7 | 0.0 ± 0.0 | 0.0 ± 0.0 | 1.0 ± 0.3 | 1.7 ± 1.1 |  | 0.049 | 0.885 |
| *Ruminococcus gnavus* | 0.0 ± 0.0 | 1.8 ± 0.7 | 1.6 ± 0.7 | 0.2 ± 0.3 | 0.1 ± 0.1 | 0.1 ± 0.1 | 2.1 ± 0.7 | 5.0 ± 4.9 | 0.0 ± 0.1 | 0.1 ± 0.1 |  | 0.059 | 0.278 |
| *Ruminococcus lactaris* | 0.6 ± 0.2 | 0.0 ± 0.0 | 0.1 ± 0.1 | 0.5 ± 0.3 | 0.1 ± 0.1 | 0.3 ± 0.2 | 0.0 ± 0.0 | 0.0 ± 0.0 | 0.1 ± 0.1 | 0.9 ± 0.3 |  | 0.029 | 0.668 |
| *Ruminococcus obeum* | 1.6 ± 0.7 | 0.2 ± 0.1 | 0.1 ± 0.1 | 1.2 ± 0.8 | 0.0 ± 0.0 | 1.1 ± 0.5 | 1.2 ± 0.2 | 0.2 ± 0.1 | 1.2 ± 0.2 | 0.7 ± 0.1 |  | 0.004 | 0.759 |
| *Ruminococcus torques* | 0.5 ± 0.4 | 0.8 ± 0.3 | 1.1 ± 0.3 | 0.2 ± 0.2 | 0.7 ± 0.3 | 0.5 ± 0.3 | 0.0 ± 0.0 | 2.4 ± 2.5 | 0.3 ± 0.4 | 0.1 ± 0.1 |  | 0.015 | 0.358 |
| *Streptococcus salivarius* | 0.5 ± 0.3 | 0.4 ± 0.4 | 0.4 ± 0.3 | 0.2 ± 0.2 | 0.1 ± 0.0 | 0.1 ± 0.0 | 0.4 ± 0.2 | 0.1 ± 0.1 | 0.4 ± 0.3 | 0.2 ± 0.2 |  | <0.001 | 0.045 |
| *Streptococcus thermophilus* | 0.5 ± 0.5 | 0.7 ± 0.9 | 0.4 ± 0.2 | 1.3 ± 0.9 | 0.5 ± 0.5 | 0.0 ± 0.0 | 0.4 ± 0.4 | 0.6 ± 1.0 | 0.0 ± 0.0 | 0.0 ± 0.0 |  | 0.006 | 0.440 |
| *Subdoligranulum variabile* | 2.2 ± 0.8 | 1.9 ± 1.0 | 3.0 ± 1.7 | 0.5 ± 0.8 | 4.6 ± 0.7 | 1.9 ± 0.5 | 0.0 ± 0.1 | 0.6 ± 0.5 | 4.2 ± 0.5 | 0.5 ± 0.2 |  | 0.004 | 0.408 |
| *Veillonella ratti* | 0.0 ± 0.0 | − | 4.4 ± 1.8 | − | 0.0 ± 0.0 | 0.0 ± 0.0 | 0.0 ± 0.0 | 0.1 ± 0.1 | 0.0 ± 0.0 | 0.0 ± 0.0 |  | 0.335 | 0.410 |
| Proteobacteria |  |  |  |  |  |  |  |  |  |  |  |  |  |
| *Parasutterella excrementihominis* | 0.0 ± 0.0 | 1.4 ± 0.5 | 0.0 ± 0.0 | 0.1 ± 0.1 | 0.0 ± 0.0 | 0.4 ± 0.1 | 0.0 ± 0.0 | 0.0 ± 0.0 | − | 0.0 ± 0.0 |  | 0.222 | 0.382 |
| *Sphingomonas leidyi* | 0.1 ± 0.1 | 0.0 ± 0.0 | 0.0 ± 0.0 | 0.5 ± 0.7 | 0.0 ± 0.0 | 0.1 ± 0.1 | 0.0 ± 0.0 | 0.0 ± 0.0 | 0.9 ± 1.2 | 0.1 ± 0.1 |  | 0.083 | 0.428 |
| *Sutterella stercoricanis* | 0.6 ± 0.1 | 0.0 ± 0.0 | 0.0 ± 0.0 | 0.1 ± 0.2 | − | 0.0 ± 0.0 | 0.0 ± 0.0 | 0.0 ± 0.0 | 0.0 ± 0.0 | 1.3 ± 0.2 |  | 0.168 | 0.218 |
| *Sutterella wadsworthensis* | 0.4 ± 0.1 | 0.0 ± 0.0 | − | 0.0 ± 0.1 | 0.7 ± 0.2 | 0.0 ± 0.0 | 0.0 ± 0.0 | − | 0.0 ± 0.1 | 0.0 ± 0.0 |  | 0.177 | 0.258 |
| Verrucomicrobia |  |  |  |  |  |  |  |  |  |  |  |  |  |
| *Akkermansia muciniphila* | 0.0 ± 0.1 | 0.0 ± 0.0 | 0.1 ± 0.2 | 1.0 ± 0.5 | 0.0 ± 0.0 | 0.3 ± 0.4 | 0.0 ± 0.0 | 0.0 ± 0.0 | 0.0 ± 0.0 | 1.1 ± 1.0 |  | 0.090 | 0.554 |
| Others | 32.2 ± 2.4 | 28.8 ± 4.4 | 33.5 ± 6.2 | 39.8 ± 4 | 29.8 ± 2.7 | 47.6 ± 7.5 | 20.4 ± 0.7 | 22.5 ± 1.4 | 60.9 ± 7.8 | 25.3 ± 1.4 |  | <0.001 | 0.817 |

^a^ The individual means ± SD was calculated using all values for 1 year.

^b^ According to one-way repeated measurement ANOVA, the residual was defined as “Season”. The two variables, “Subject” and “Season”, correspond to “inter-” and “intra-” individual variations, respectively.

^c^ Not detected.
