# Supplementary material for: Inter- and intra-individual variations in seasonal and daily stabilities of the human gut microbiota in Japanese
Source: Arch Microbiol. 2015 Jun 12;197(7):919–34. doi: 10.1007/s00203-015-1125-0 (PMC4536265; doi:10.1007/s00203-015-1125-0)
Supplement: Supplementary file 2 — Supplementary material 2 (DOCX 44 kb) [file 203_2015_1125_MOESM2_ESM.docx]

**Table S2** Compositions (%) of 83 selected dominant species in the human gut microbiota of the 10 subjects (S1–S10), based on daily stability

| Phylum | Subject (S1–S10) | | | | | | | | | |  | *P* value^b^ | |
| --- | --- | --- | --- | --- | --- | --- | --- | --- | --- | --- | --- | --- | --- |
| *Species* | S1 | S2 | S3 | S4 | S5 | S6 | S7 | S8 | S9 | S10 |  | Subject | Day |
| Actinobacteria |  |  |  |  |  |  |  |  |  |  |  |  |  |
| *Bifidobacterium longum* | 0.6 ± 0.2^a^ | 2.4 ± 0.7 | 4.3 ± 1.7 | 0.8 ± 0.2 | 0.0 ± 0.0 | 0.0 ± 0.0 | 0.0 ± 0.0 | 1.0 ± 0.5 | 0.2 ± 0.1 | 1.0 ± 0.3 |  | 0.039 | 0.478 |
| *Collinsella aerofaciens* | 4.8 ± 1.2 | 5.9 ± 1.1 | 0.1 ± 0.1 | 0.0 ± 0.0 | 3.2 ± 0.4 | 4.5 ± 0.4 | 0.1 ± 0.0 | 5.0 ± 1.9 | 3.3 ± 1.4 | 3.0 ± 0.7 |  | 0.002 | 0.161 |
| *Eggerthella lenta* | −^c^ | 0.1 ± 0.0 | 0.1 ± 0.1 | 0.1 ± 0.0 | 0.1 ± 0.0 | 0.0 ± 0.0 | 0.3 ± 0.1 | 0.3 ± 0.2 | − | 0.1 ± 0.0 |  | 0.011 | 0.208 |
| Bacteroidetes |  |  |  |  |  |  |  |  |  |  |  |  |  |
| *Alistipes onderdonkii* | 0.1 ± 0.1 | 0.0 ± 0.0 | 0.1 ± 0.1 | 0.2 ± 0.1 | 0.0 ± 0.0 | 1.7 ± 0.8 | 0.0 ± 0.0 | 0.1 ± 0.1 | 0.0 ± 0.0 | 0.7 ± 0.4 |  | 0.141 | 0.674 |
| *Alistipes putredinis* | 0.2 ± 0.2 | 0.6 ± 0.3 | 0.0 ± 0.0 | 0.8 ± 0.2 | 0.5 ± 0.1 | 0.1 ± 0.0 | 0.0 ± 0.0 | 0.2 ± 0.4 | 0.0 ± 0.0 | 0.1 ± 0.1 |  | 0.033 | 0.736 |
| *Alistipes shahii* | 0.1 ± 0.1 | 0.0 ± 0.0 | − | 0.6 ± 0.3 | 0.5 ± 0.1 | 0.6 ± 0.2 | 0.0 ± 0.0 | 0.1 ± 0.1 | 0.0 ± 0.0 | 0.0 ± 0.0 |  | 0.061 | 0.756 |
| *Bacteroides clarus* | 0.0 ± 0.0 | 0.3 ± 0.1 | 0.1 ± 0.1 | 0.0 ± 0.0 | − | − | − | − | 0.0 ± 0.0 | − |  | 0.226 | 0.852 |
| *Bacteroides coprocola* | 3.3 ± 0.8 | 0.0 ± 0.0 | 0.0 ± 0.0 | 0.0 ± 0.0 | 0.0 ± 0.0 | 0.0 ± 0.0 | 0.0 ± 0.0 | 0.9 ± 2.0 | 0.0 ± 0.0 | 0.2 ± 0.6 |  | 0.235 | 0.437 |
| *Bacteroides dorei* | 0.1 ± 0.1 | 1.8 ± 0.4 | 0.1 ± 0.1 | 0.0 ± 0.0 | 6.3 ± 1.0 | 1.9 ± 1.9 | 2.6 ± 0.4 | 3.0 ± 2.0 | 0.0 ± 0.0 | 0.0 ± 0.0 |  | 0.037 | 0.233 |
| *Bacteroides eggerthii* | 0.0 ± 0.0 | 0.5 ± 0.3 | − | − | − | 0.1 ± 0.0 | 0.0 ± 0.0 | 0.0 ± 0.0 | 0.0 ± 0.0 | 0.0 ± 0.0 |  | 0.258 | 0.435 |
| *Bacteroides faecichinchillae* | 0.0 ± 0.0 | 0.4 ± 0.2 | 0.0 ± 0.0 | 0.1 ± 0.0 | 0.2 ± 0.0 | 0.5 ± 0.2 | 1.1 ± 0.4 | 0.1 ± 0.1 | 0.0 ± 0.0 | 0.0 ± 0.0 |  | 0.077 | 0.658 |
| *Bacteroides faecis* | 0.1 ± 0.1 | 0.0 ± 0.0 | 0.0 ± 0.0 | 0.0 ± 0.0 | 0.0 ± 0.0 | 0.1 ± 0.1 | 1.8 ± 0.6 | 0.7 ± 0.7 | 0.0 ± 0.0 | 0.2 ± 0.1 |  | 0.140 | 0.689 |
| *Bacteroides finegoldii* | 0.1 ± 0.1 | 0.0 ± 0.0 | − | 0.0 ± 0.0 | 0.0 ± 0.0 | 0.1 ± 0.0 | 0.0 ± 0.0 | 0.1 ± 0.2 | 0.0 ± 0.0 | 1.1 ± 0.4 |  | 0.240 | 0.619 |
| *Bacteroides fragilis* | 0.0 ± 0.0 | 0.1 ± 0.1 | 0.1 ± 0.0 | 0.6 ± 0.5 | 0.0 ± 0.0 | 0.0 ± 0.0 | 0.0 ± 0.0 | 0.7 ± 0.6 | 0.0 ± 0.0 | 0.1 ± 0.0 |  | 0.127 | 0.712 |
| *Bacteroides massiliensis* | 1.5 ± 1.1 | 3.3 ± 1.0 | 0.0 ± 0.0 | 0.0 ± 0.0 | − | 0.0 ± 0.0 | 0.0 ± 0.0 | 0.0 ± 0.0 | 0.0 ± 0.0 | 0.0 ± 0.1 |  | 0.196 | 0.817 |
| *Bacteroides ovatus* | 0.1 ± 0.1 | 0.0 ± 0.0 | 0.4 ± 0.3 | 0.4 ± 0.2 | 1.4 ± 0.6 | 0.2 ± 0.1 | 6.1 ± 1.6 | 0.1 ± 0.1 | 0.0 ± 0.0 | 0.6 ± 0.3 |  | 0.152 | 0.312 |
| *Bacteroides plebeius* | 0.0 ± 0.0 | 0.0 ± 0.0 | 9.0 ± 2.7 | 0.0 ± 0.0 | 0.0 ± 0.0 | 3.3 ± 0.9 | 0.0 ± 0.0 | 10.9 ± 5.4 | 0.0 ± 0.0 | 0.0 ± 0.0 |  | 0.112 | 0.571 |
| *Bacteroides stercoris* | 0.2 ± 0.1 | 0.0 ± 0.0 | 0.0 ± 0.0 | 0.0 ± 0.0 | 1.1 ± 0.4 | 0.1 ± 0.0 | 0.0 ± 0.0 | 0.0 ± 0.1 | 0.0 ± 0.0 | 0.0 ± 0.0 |  | 0.207 | 0.511 |
| *Bacteroides uniformis* | 0.8 ± 1.0 | 1.9 ± 0.7 | 0.2 ± 0.2 | 3.7 ± 1.6 | 0.8 ± 0.1 | 1.2 ± 0.2 | 9.0 ± 2.7 | 1.7 ± 0.6 | 0.1 ± 0.1 | 0.1 ± 0.2 |  | 0.051 | 0.501 |
| *Bacteroides vulgatus* | 2.3 ± 1.5 | 8.2 ± 1.7 | 8.0 ± 3.9 | 0.0 ± 0.0 | 0.0 ± 0.0 | 0.7 ± 0.5 | 6.5 ± 0.4 | 0.8 ± 1.5 | 0.1 ± 0.1 | 10.2 ± 2.1 |  | 0.020 | 0.411 |
| *Bacteroides xylanisolvens* | 0.0 ± 0.0 | 0.7 ± 0.4 | 0.2 ± 0.1 | 1.3 ± 0.6 | 0.0 ± 0.0 | 0.1 ± 0.0 | 0.1 ± 0.0 | 0.5 ± 0.3 | 0.0 ± 0.0 | 1.3 ± 0.4 |  | 0.039 | 0.332 |
| *Barnesiella intestinihominis* | 0.3 ± 0.3 | 0.0 ± 0.0 | 0.0 ± 0.0 | 0.7 ± 0.1 | 0.0 ± 0.0 | 0.1 ± 0.0 | 0.0 ± 0.0 | 0.1 ± 0.1 | 0.0 ± 0.0 | 0.0 ± 0.0 |  | 0.123 | 0.704 |
| *Odoribacter splanchnicus* | 0.1 ± 0.1 | − | 0.0 ± 0.0 | − | 0.2 ± 0.0 | 0.3 ± 0.0 | 0.0 ± 0.0 | 0.0 ± 0.1 | 0.0 ± 0.0 | 0.2 ± 0.1 |  | 0.031 | 0.777 |
| *Parabacteroides distasonis* | 0.1 ± 0.1 | 1.3 ± 0.8 | 0.7 ± 0.4 | 2.0 ± 0.5 | 0.0 ± 0.0 | 0.3 ± 0.1 | 0.0 ± 0.0 | 0.7 ± 0.2 | 0.3 ± 0.1 | 0.0 ± 0.1 |  | 0.031 | 0.435 |
| *Parabacteroides johnsonii* | − | − | − | − | − | − | − | 0.0 ± 0.0 | 0.0 ± 0.0 | 1.1 ± 0.7 |  | 0.343 | 0.435 |
| *Parabacteroides merdae* | 0.4 ± 0.2 | 0.0 ± 0.0 | 0.0 ± 0.0 | 0.0 ± 0.0 | − | 0.9 ± 0.7 | 0.0 ± 0.0 | 1.0 ± 0.5 | 0.0 ± 0.0 | 0.0 ± 0.0 |  | 0.104 | 0.152 |
| *Prevotella copri* | 7.7 ± 2.4 | 0.0 ± 0.0 | 0.5 ± 0.5 | 0.1 ± 0.0 | 0.0 ± 0.0 | 2.2 ± 2.6 | 0.4 ± 0.2 | 0.1 ± 0.1 | 9.4 ± 5.8 | 0.6 ± 1.3 |  | 0.101 | 0.364 |
| *Prevotella stercorea* | − | 0.0 ± 0.0 | 0.0 ± 0.0 | 0.0 ± 0.0 | − | − | 0.0 ± 0.0 | 0.0 ± 0.0 | 1.6 ± 1.2 | 0.1 ± 0.3 |  | 0.301 | 0.489 |

to be continued

**Table S2** continued

| Phylum | Subject (S1–S10) | | | | | | | | | |  | *P* value | |
| --- | --- | --- | --- | --- | --- | --- | --- | --- | --- | --- | --- | --- | --- |
| *Species* | S1 | S2 | S3 | S4 | S5 | S6 | S7 | S8 | S9 | S10 |  | Subject | Day |
| Firmicutes |  |  |  |  |  |  |  |  |  |  |  |  |  |
| *Blautia faecis* | 0.3 ± 0.2 | 0.3 ± 0.0 | 0.4 ± 0.3 | 2.7 ± 0.6 | 1.1 ± 0.1 | 0.4 ± 0.1 | 0.4 ± 0.2 | 0.7 ± 0.4 | 0.5 ± 0.3 | 1.0 ± 0.2 |  | 0.008 | 0.070 |
| *Blautia glucerasea* | 0.0 ± 0.0 | 0.4 ± 0.1 | 0.2 ± 0.1 | 0.1 ± 0.0 | 0.0 ± 0.0 | 0.0 ± 0.0 | 0.1 ± 0.0 | 0.4 ± 0.2 | 0.0 ± 0.0 | 0.0 ± 0.0 |  | 0.051 | 0.403 |
| *Blautia luti* | 2.7 ± 1.8 | 2.7 ± 0.6 | 12.8 ± 2.3 | 3.2 ± 0.3 | 8.5 ± 0.7 | 3.4 ± 0.8 | 0.1 ± 0.0 | 2.6 ± 1.4 | 1.5 ± 0.7 | 5.5 ± 0.8 |  | 0.005 | 0.521 |
| *Blautia stercoris* | 0.6 ± 0.6 | 1.3 ± 0.4 | 0.0 ± 0.0 | 0.0 ± 0.0 | 0.0 ± 0.0 | 0.0 ± 0.1 | 0.4 ± 0.1 | 0.0 ± 0.0 | 0.1 ± 0.1 | 0.0 ± 0.0 |  | 0.113 | 0.376 |
| *Blautia wexlerae* | 1.6 ± 0.6 | 7.9 ± 0.8 | 4.2 ± 1.0 | 8.8 ± 0.5 | 5.2 ± 1.2 | 2.0 ± 0.8 | 13.9 ± 2.1 | 10.4 ± 3.7 | 4.4 ± 1.4 | 8.0 ± 1.4 |  | <0.001 | 0.359 |
| *Catenibacterium mitsuokai* | 0.0 ± 0.0 | − | 0.0 ± 0.0 | 0.2 ± 0.0 | − | 0.0 ± 0.0 | − | 0.0 ± 0.0 | 0.6 ± 0.2 | 0.0 ± 0.1 |  | 0.241 | 0.403 |
| *Clostridium bartlettii* | 0.3 ± 0.3 | 0.3 ± 0.1 | 0.2 ± 0.2 | 0.0 ± 0.0 | 0.2 ± 0.0 | 0.1 ± 0.2 | 0.4 ± 0.1 | 0.1 ± 0.2 | 0.0 ± 0.0 | 0.1 ± 0.0 |  | 0.005 | 0.244 |
| *Clostridium celerecrescens* | − | − | 0.0 ± 0.0 | − | 0.0 ± 0.0 | 0.0 ± 0.0 | 0.0 ± 0.0 | 0.4 ± 0.3 | − | 0.0 ± 0.0 |  | 0.343 | 0.435 |
| *Clostridium disporicum* | 0.8 ± 0.5 | 0.1 ± 0.1 | 0.4 ± 0.3 | 0.3 ± 0.1 | 0.3 ± 0.2 | 0.1 ± 0.1 | 0.0 ± 0.0 | 0.0 ± 0.1 | 0.0 ± 0.0 | 0.0 ± 0.0 |  | 0.038 | 0.536 |
| *Clostridium leptum* | 0.0 ± 0.0 | 0.1 ± 0.1 | 0.0 ± 0.0 | 0.0 ± 0.0 | 0.1 ± 0.0 | 0.1 ± 0.0 | 1.1 ± 0.6 | 0.0 ± 0.0 | 0.0 ± 0.0 | 0.0 ± 0.0 |  | 0.239 | 0.798 |
| *Clostridium lituseburense* | 0.5 ± 0.1 | 0.6 ± 0.6 | 0.1 ± 0.0 | 0.2 ± 0.1 | 0.1 ± 0.0 | 0.6 ± 0.3 | 0.1 ± 0.0 | 0.2 ± 0.1 | 0.1 ± 0.1 | 0.1 ± 0.0 |  | 0.010 | 0.482 |
| *Clostridium xylanolyticum* | 0.0 ± 0.0 | 0.0 ± 0.0 | 0.0 ± 0.0 | 1.1 ± 0.3 | 0.0 ± 0.0 | 0.2 ± 0.1 | 0.0 ± 0.0 | 0.0 ± 0.1 | 0.1 ± 0.1 | 0.1 ± 0.0 |  | 0.206 | 0.284 |
| *Coprococcus catus* | 0.3 ± 0.1 | 0.0 ± 0.0 | 0.0 ± 0.0 | 0.5 ± 0.1 | 0.0 ± 0.0 | 0.7 ± 0.1 | 0.0 ± 0.0 | 0.1 ± 0.2 | 0.5 ± 0.2 | 0.6 ± 0.1 |  | 0.016 | 0.216 |
| *Coprococcus comes* | 0.5 ± 0.1 | 0.0 ± 0.0 | 0.8 ± 0.1 | 0.0 ± 0.0 | 2.1 ± 0.2 | 0.5 ± 0.1 | 1.0 ± 0.2 | 0.1 ± 0.1 | 1.1 ± 0.3 | 0.2 ± 0.2 |  | 0.017 | 0.908 |
| *Coprococcus eutactus* | 0.6 ± 0.5 | 0.0 ± 0.0 | 0.1 ± 0.2 | 0.0 ± 0.0 | 0.5 ± 0.1 | 0.0 ± 0.0 | − | 0.5 ± 1.0 | 0.4 ± 0.2 | 0.1 ± 0.3 |  | 0.030 | 0.378 |
| *Dialister succinatiphilus* | − | − | − | 0.0 ± 0.0 | − | 0.0 ± 0.0 | − | 1.0 ± 0.5 | 0.0 ± 0.0 | 0.0 ± 0.0 |  | 0.343 | 0.435 |
| *Dorea formicigenerans* | 0.3 ± 0.1 | 0.0 ± 0.0 | 0.2 ± 0.1 | 0.0 ± 0.0 | 0.0 ± 0.0 | 0.2 ± 0.1 | 0.0 ± 0.0 | 0.5 ± 0.1 | 0.2 ± 0.1 | 0.3 ± 0.0 |  | 0.010 | 0.147 |
| *Dorea longicatena* | 1.3 ± 0.4 | 1.5 ± 0.2 | 1.8 ± 0.3 | 0.0 ± 0.0 | 0.0 ± 0.0 | 0.7 ± 0.1 | 2.5 ± 0.4 | 1.9 ± 0.3 | 0.7 ± 0.1 | 0.8 ± 0.1 |  | 0.002 | 0.744 |
| *Eubacterium coprostanoligenes* | − | − | 0.0 ± 0.0 | 0.5 ± 0.1 | 0.0 ± 0.0 | 0.0 ± 0.0 | 0.0 ± 0.0 | 0.1 ± 0.3 | 0.4 ± 0.0 | 0.0 ± 0.1 |  | 0.090 | 0.614 |
| *Eubacterium desmolans* | 0.0 ± 0.0 | 0.8 ± 0.2 | 0.0 ± 0.0 | 0.0 ± 0.0 | 0.0 ± 0.0 | 0.0 ± 0.0 | 0.0 ± 0.0 | 0.0 ± 0.1 | 0.2 ± 0.0 | 0.0 ± 0.1 |  | 0.220 | 0.188 |
| *Eubacterium eligens* | 0.0 ± 0.0 | 0.0 ± 0.0 | 0.0 ± 0.1 | 2.3 ± 0.3 | 0.0 ± 0.0 | 0.0 ± 0.0 | 1.4 ± 0.8 | 0.1 ± 0.2 | 0.1 ± 0.1 | 0.0 ± 0.1 |  | 0.156 | 0.418 |
| *Eubacterium hadrum* | 0.3 ± 0.1 | 2.2 ± 0.7 | 5.2 ± 2.7 | 3.1 ± 0.5 | 0.0 ± 0.0 | 1.1 ± 0.3 | 6.3 ± 0.8 | 0.3 ± 0.4 | 0.4 ± 0.1 | 3.9 ± 1.1 |  | 0.012 | 0.548 |
| *Eubacterium hallii* | 1.0 ± 0.7 | 2.4 ± 0.4 | 1.1 ± 0.1 | 1.3 ± 0.2 | 2.2 ± 0.4 | 1.0 ± 0.2 | 0.1 ± 0.0 | 1.9 ± 1.1 | 2.0 ± 1.0 | 2.0 ± 0.2 |  | <0.001 | 0.290 |
| *Eubacterium ramulus* | 0.2 ± 0.1 | − | 0.2 ± 0.1 | − | 0.4 ± 0.1 | 0.2 ± 0.0 | 0.0 ± 0.0 | 0.0 ± 0.0 | 0.1 ± 0.0 | 0.1 ± 0.0 |  | 0.017 | 0.491 |
| *Eubacterium rectale* | 1.2 ± 0.7 | 0.0 ± 0.0 | 0.5 ± 0.2 | 0.1 ± 0.0 | 0.0 ± 0.0 | 0.0 ± 0.0 | 0.0 ± 0.0 | 0.1 ± 0.1 | 0.8 ± 0.6 | 2.2 ± 0.3 |  | 0.073 | 0.424 |
| *Eubacterium ruminantium* | 0.5 ± 0.7 | 0.0 ± 0.0 | 0.0 ± 0.0 | 0.0 ± 0.0 | − | 1.7 ± 0.3 | 0.0 ± 0.0 | 0.1 ± 0.2 | 0.0 ± 0.0 | 0.0 ± 0.0 |  | 0.220 | 0.555 |
| *Eubacterium siraeum* | 0.0 ± 0.0 | − | 0.0 ± 0.0 | 0.8 ± 0.7 | 0.1 ± 0.1 | 1.6 ± 1.2 | 0.0 ± 0.0 | 0.0 ± 0.0 | 0.8 ± 0.7 | 0.0 ± 0.1 |  | 0.089 | 0.358 |
| *Eubacterium ventriosum* | 0.2 ± 0.1 | 0.5 ± 0.2 | 0.3 ± 0.2 | 0.2 ± 0.1 | 0.6 ± 0.1 | 0.2 ± 0.1 | 1.0 ± 0.2 | 0.0 ± 0.0 | 0.0 ± 0.0 | 0.3 ± 0.1 |  | 0.008 | 0.673 |
| *Faecalibacterium prausnitzii* | 4.4 ± 1.4 | 4.7 ± 1.1 | 3.9 ± 2.1 | 8.2 ± 2.6 | 6.0 ± 3.4 | 6.1 ± 2.3 | 12.7 ± 3.6 | 5.0 ± 2.1 | 6.5 ± 1.6 | 10.4 ± 1.3 |  | <0.001 | 0.720 |
| *Lachnospira pectinoschiza* | 0.0 ± 0.0 | 2.7 ± 0.8 | 0.0 ± 0.0 | 0.0 ± 0.0 | 0.0 ± 0.0 | 0.1 ± 0.0 | − | 0.1 ± 0.0 | 0.0 ± 0.0 | 0.0 ± 0.0 |  | 0.325 | 0.446 |
| *Megamonas funiformis* | 15.8 ± 8.0 | 0.0 ± 0.0 | 0.0 ± 0.0 | 0.0 ± 0.0 | 0.0 ± 0.0 | 0.0 ± 0.0 | 0.1 ± 0.1 | 10.8 ± 7.7 | 0.7 ± 0.7 | 0.1 ± 0.1 |  | 0.163 | 0.740 |
| *Megamonas rupellensis* | 0.0 ± 0.0 | − | − | 0.0 ± 0.0 | − | − | 0.0 ± 0.0 | 0.2 ± 0.1 | 0.9 ± 0.0 | 0.0 ± 0.1 |  | 0.226 | 0.517 |

to be continued

**Table S2** continued

| Phylum | Subject (S1–S10) | | | | | | | | | |  | *P* value | |
| --- | --- | --- | --- | --- | --- | --- | --- | --- | --- | --- | --- | --- | --- |
| *Species* | S1 | S2 | S3 | S4 | S5 | S6 | S7 | S8 | S9 | S10 |  | Subject | Day |
| *Megasphaera elsdenii* | 3.0 ± 1.8 | 0.0 ± 0.0 | 0.0 ± 0.0 | − | − | 0.5 ± 0.1 | 0.0 ± 0.0 | 3.9 ± 3.2 | 0.0 ± 0.0 | 0.0 ± 0.0 |  | 0.141 | 0.587 |
| *Mitsuokella multacida* | − | 0.0 ± 0.0 | 0.0 ± 0.0 | − | − | 0.9 ± 0.6 | − | 0.0 ± 0.0 | 2.4 ± 0.0 | 0.0 ± 0.1 |  | 0.210 | 0.446 |
| *Phascolarctobacterium faecium* | 0.0 ± 0.0 | 0.0 ± 0.0 | 0.0 ± 0.0 | 0.6 ± 0.1 | 0.0 ± 0.0 | 0.0 ± 0.0 | 1.8 ± 0.5 | 0.0 ± 0.0 | 0.0 ± 0.0 | 0.7 ± 0.2 |  | 0.126 | 0.612 |
| *Phascolarctobacterium succinatutens* | 0.0 ± 0.0 | 0.8 ± 0.1 | 0.0 ± 0.0 | 0.0 ± 0.0 | − | 0.0 ± 0.0 | 0.0 ± 0.0 | 0.1 ± 0.3 | 0.4 ± 0.2 | 0.1 ± 0.1 |  | 0.152 | 0.952 |
| *Roseburia faecis* | 0.1 ± 0.1 | 2.1 ± 0.5 | 0.1 ± 0.1 | 2.2 ± 0.6 | 0.0 ± 0.0 | 0.6 ± 0.5 | 0.2 ± 0.1 | 1.3 ± 0.8 | 0.0 ± 0.0 | 0.2 ± 0.1 |  | 0.040 | 0.535 |
| *Roseburia intestinalis* | 0.0 ± 0.0 | 1.4 ± 0.5 | 0.1 ± 0.1 | 0.0 ± 0.0 | 1.1 ± 1.1 | 0.0 ± 0.0 | 0.0 ± 0.0 | 0.1 ± 0.1 | 0.5 ± 0.4 | 0.1 ± 0.2 |  | 0.091 | 0.721 |
| *Roseburia inulinivorans* | 0.0 ± 0.0 | 1.1 ± 0.2 | 0.0 ± 0.0 | − | 0.0 ± 0.0 | 0.3 ± 0.2 | 0.5 ± 0.2 | 0.0 ± 0.0 | 0.5 ± 0.3 | 0.5 ± 0.3 |  | 0.032 | 0.791 |
| *Ruminococcus bromii* | 0.3 ± 0.2 | 0.0 ± 0.0 | 0.1 ± 0.1 | 5.3 ± 1.4 | 4.2 ± 0.2 | 2.9 ± 0.9 | 0.0 ± 0.0 | 0.2 ± 0.3 | 0.5 ± 0.5 | 0.1 ± 0.1 |  | 0.065 | 0.321 |
| *Ruminococcus callidus* | 1.0 ± 0.6 | 0.0 ± 0.0 | 0.0 ± 0.0 | 0.0 ± 0.0 | 1.0 ± 0.5 | 0.3 ± 0.2 | − | 0.0 ± 0.1 | 0.1 ± 0.2 | 0.7 ± 0.3 |  | 0.041 | 0.874 |
| *Ruminococcus faecis* | 1.3 ± 0.4 | 0.0 ± 0.0 | 0.1 ± 0.1 | 0.0 ± 0.0 | 0.0 ± 0.0 | 4.3 ± 0.3 | 0.1 ± 0.0 | 0.2 ± 0.5 | 1.7 ± 0.8 | 1.0 ± 0.2 |  | 0.080 | 0.210 |
| *Ruminococcus gnavus* | 0.0 ± 0.0 | 2.3 ± 1.2 | 2.2 ± 0.8 | 0.1 ± 0.1 | 0.1 ± 0.0 | 0.0 ± 0.0 | 1.5 ± 0.2 | 0.9 ± 0.5 | 0.0 ± 0.0 | 0.0 ± 0.0 |  | 0.045 | 0.240 |
| *Ruminococcus lactaris* | 0.6 ± 0.2 | 0.0 ± 0.0 | 0.0 ± 0.0 | 1.0 ± 0.1 | 0.0 ± 0.0 | 0.3 ± 0.2 | 0.0 ± 0.0 | 0.1 ± 0.2 | 0.3 ± 0.3 | 1.1 ± 0.2 |  | 0.037 | 0.731 |
| *Ruminococcus obeum* | 1.1 ± 0.8 | 0.2 ± 0.1 | 0.0 ± 0.0 | 1.4 ± 0.1 | 0.0 ± 0.0 | 0.8 ± 0.2 | 1.8 ± 0.6 | 0.5 ± 0.2 | 0.6 ± 0.1 | 0.6 ± 0.1 |  | 0.004 | 0.495 |
| *Ruminococcus torques* | 0.2 ± 0.3 | 0.9 ± 0.4 | 0.7 ± 0.6 | 0.2 ± 0.2 | 0.7 ± 0.2 | 0.7 ± 0.7 | 0.0 ± 0.0 | 1.0 ± 0.6 | 0.0 ± 0.0 | 0.0 ± 0.0 |  | 0.006 | 0.475 |
| *Streptococcus salivarius* | 0.6 ± 0.1 | 0.4 ± 0.2 | 1.4 ± 0.4 | 0.2 ± 0.1 | 0.1 ± 0.0 | 0.0 ± 0.0 | 0.4 ± 0.2 | 0.1 ± 0.1 | 0.4 ± 0.2 | 0.2 ± 0.0 |  | 0.022 | 0.434 |
| *Streptococcus thermophilus* | 1.9 ± 2.1 | 0.0 ± 0.0 | 1.0 ± 0.9 | 1.1 ± 0.4 | 1.1 ± 0.5 | 0.0 ± 0.0 | 0.0 ± 0.0 | 0.0 ± 0.0 | 0.0 ± 0.0 | 0.2 ± 0.3 |  | 0.036 | 0.135 |
| *Subdoligranulum variabile* | 1.3 ± 0.6 | 2.8 ± 0.6 | 1.8 ± 0.6 | 0.0 ± 0.0 | 6.4 ± 0.7 | 2.3 ± 0.3 | 0.0 ± 0.0 | 1.9 ± 1.0 | 3.7 ± 0.2 | 1.1 ± 0.4 |  | 0.007 | 0.603 |
| *Veillonella ratti* | 0.0 ± 0.0 | 0.0 ± 0.0 | 3.4 ± 1.5 | 0.0 ± 0.0 | − | − | 0.0 ± 0.0 | 0.0 ± 0.1 | 0.0 ± 0.0 | 0.0 ± 0.0 |  | 0.339 | 0.481 |
| Proteobacteria |  |  |  |  |  |  |  |  |  |  |  |  |  |
| *Parasutterella excrementihominis* | 0.0 ± 0.0 | 1.6 ± 0.3 | 0.0 ± 0.0 | 0.0 ± 0.0 | − | 0.4 ± 0.1 | − | 0.0 ± 0.0 | 0.0 ± 0.0 | 0.0 ± 0.0 |  | 0.240 | 0.387 |
| *Sphingomonas leidyi* | 0.0 ± 0.0 | 0.7 ± 0.2 | 1.3 ± 1.0 | 0.3 ± 0.1 | 0.1 ± 0.0 | 0.3 ± 0.2 | 1.3 ± 0.5 | 1.4 ± 1.8 | 1.1 ± 0.5 | 1.6 ± 1.4 |  | 0.003 | 0.397 |
| *Sutterella stercoricanis* | 0.8 ± 0.2 | 0.0 ± 0.0 | 0.0 ± 0.0 | − | − | − | − | 0.1 ± 0.2 | 0.1 ± 2.0 | 1.2 ± 0.4 |  | 0.144 | 0.202 |
| *Sutterella wadsworthensis* | 0.2 ± 0.1 | 0.0 ± 0.0 | − | 0.0 ± 0.0 | 0.6 ± 0.3 | 0.0 ± 0.0 | 0.0 ± 0.0 | 0.0 ± 0.0 | 0.0 ± 0.0 | 0.0 ± 0.0 |  | 0.205 | 0.156 |
| Verrucomicrobia |  |  |  |  |  |  |  |  |  |  |  |  |  |
| *Akkermansia muciniphila* | 0.0 ± 0.0 | − | 0.0 ± 0.0 | 0.4 ± 0.1 | 8.0 ± 5.9 | 0.0 ± 0.0 | 0.0 ± 0.0 | 0.2 ± 0.5 | 0.1 ± 0.1 | 0.7 ± 0.6 |  | 0.270 | 0.438 |
| Others | 31.1 ± 2.9 | 29.6 ± 3.2 | 31.3 ± 6.8 | 42.3 ± 2.2 | 34.9 ± 3.3 | 45.0 ± 6.0 | 22.4 ± 3.1 | 22.0 ± 10.2 | 49.1 ± 6.0 | 32.9 ± 3.2 |  | <0.001 | 0.443 |

^a^ The individual means ± SD was calculated using all values for 1 week.

^b^ According to one-way repeated measurement ANOVA, the residual was defined as “Day”. The two variables, “Subject” and “Day”, correspond to “inter-” and “intra-” individual variations, respectively.

^c^ Not detected.
