# Supplementary material for: Inter- and intra-individual variations in seasonal and daily stabilities of the human gut microbiota in Japanese
Source: Arch Microbiol. 2015 Jun 12;197(7):919–34. doi: 10.1007/s00203-015-1125-0 (PMC4536265; doi:10.1007/s00203-015-1125-0)
Supplement: Supplementary file 3 — Supplementary material 3 (DOCX 38 kb) [file 203_2015_1125_MOESM3_ESM.docx]

**TableS3** Relative contributions of intra- and inter-individual variance in 83 selected dominant species, coefficient of within-person variance (CV_w_) and between-person variance (CV_b_), and the number of days (“Days”) of fecal sample collection required to estimate the true values within 10 and 20 % of their true mean, based on seasonal stability

| Phylum  *Species* | Percentage contributions of variance components^a^ | | *A*/*B* | Mean^b^ | CV_w_ | CV_b_ | Days^c^ | |
| --- | --- | --- | --- | --- | --- | --- | --- | --- |
|  | Intra-individual　(*A*) | Inter-individual　(*B*) |  | (%) | (%) | (%) | 10% | 20% |
| Actinobacteria |  |  |  |  |  |  |  |  |
| *Bifidobacterium longum* | 67.9 | 32.1 | 2.1 | 1.2 | 89.4 | 151.7 | 307 | 77 |
| *Collinsellaaerofaciens* | 23.5 | 76.5 | 0.3 | 2.5 | 61.3 | 79.1 | 145 | 36 |
| *Eggerthellalenta* | 27.6 | 72.4 | 0.4 | 0.1 | 53.2 | 119.6 | 109 | 27 |
| Bacteroidetes |  |  |  |  |  |  |  |  |
| *Alistipesonderdonkii* | 27.6 | 72.4 | 0.4 | 0.3 | 74.8 | 152.7 | 215 | 54 |
| *Alistipesputredinis* | 26.7 | 73.3 | 0.4 | 0.3 | 86.6 | 128.5 | 288 | 72 |
| *Alistipesshahii* | 26.0 | 74.0 | 0.4 | 0.2 | 74.7 | 137.0 | 214 | 54 |
| *Bacteroides clarus* | 65.7 | 34.3 | 1.9 | 0.1 | 109.9 | 325.5 | 464 | 116 |
| *Bacteroides coprocola* | 46.4 | 53.6 | 0.9 | 0.6 | 130.2 | 255.6 | 651 | 163 |
| *Bacteroides dorei* | 9.9 | 90.1 | 0.1 | 2.7 | 66.9 | 133.5 | 172 | 43 |
| *Bacteroides eggerthii* | 46.5 | 53.5 | 0.9 | 0.1 | 88.3 | 256.5 | 300 | 75 |
| *Bacteroides faecichinchillae* | 30.8 | 69.2 | 0.4 | 0.3 | 88.4 | 148.7 | 300 | 75 |
| *Bacteroides faecis* | 21.4 | 78.6 | 0.3 | 0.3 | 71.4 | 196.5 | 196 | 49 |
| *Bacteroides finegoldii* | 18.9 | 81.1 | 0.2 | 0.2 | 73.7 | 241.1 | 209 | 52 |
| *Bacteroides fragilis* | 85.4 | 14.6 | 5.9 | 0.2 | 109.4 | 246.0 | 459 | 115 |
| *Bacteroides massiliensis* | 25.1 | 74.9 | 0.3 | 0.5 | 113.1 | 234.1 | 491 | 123 |
| *Bacteroides ovatus* | 10.5 | 89.5 | 0.1 | 1.2 | 59.8 | 188.0 | 137 | 34 |
| *Bacteroides plebeius* | 24.5 | 75.5 | 0.3 | 2.3 | 81.8 | 194.1 | 257 | 64 |
| *Bacteroides stercoris* | 43.4 | 56.6 | 0.8 | 0.3 | 97.6 | 183.8 | 366 | 91 |
| *Bacteroides uniformis* | 17.7 | 82.3 | 0.2 | 2.1 | 50.5 | 116.0 | 98 | 25 |
| *Bacteroides vulgatus* | 13.3 | 86.7 | 0.2 | 5.3 | 70.9 | 118.8 | 193 | 48 |
| *Bacteroides xylanisolvens* | 40.0 | 60.0 | 0.7 | 0.6 | 67.4 | 133.6 | 174 | 44 |
| *Barnesiellaintestinihominis* | 29.1 | 70.9 | 0.4 | 0.1 | 101.8 | 216.2 | 398 | 100 |
| *Odoribactersplanchnicus* | 56.1 | 43.9 | 1.3 | 0.1 | 97.7 | 110.6 | 366 | 92 |
| *Parabacteroides distasonis* | 74.4 | 25.6 | 2.9 | 0.9 | 82.9 | 175.2 | 264 | 66 |
| *Parabacteroides johnsonii* | 69.6 | 30.4 | 2.3 | 0.2 | 128.1 | 420.8 | 630 | 157 |
| *Parabacteroides merdae* | 32.0 | 68.0 | 0.5 | 0.2 | 113.1 | 160.5 | 492 | 123 |
| *Prevotella copri* | 39.4 | 60.6 | 0.7 | 0.9 | 107.8 | 239.5 | 447 | 112 |
| *Prevotella stercorea* | 56.8 | 43.2 | 1.3 | 0.0 | 149.4 | 319.5 | 857 | 214 |

to be continued

**Table S3**continued

| Phylum  *Species* | Percentage contributions of variance components^a^ | | *A*/*B* | Mean^b^ | CV_w_ | CV_b_ | Days^c^ | |
| --- | --- | --- | --- | --- | --- | --- | --- | --- |
|  | Intra-individual　(*A*) | Inter-individual　(*B*) |  | (%) | (%) | (%) | 10% | 20% |
| Firmicutes |  |  |  |  |  |  |  |  |
| *Blautia faecis* | 71.1 | 28.9 | 2.5 | 0.8 | 47.0 | 104.6 | 85 | 21 |
| *Blautia glucerasea* | 67.9 | 32.1 | 2.1 | 0.2 | 79.4 | 225.2 | 242 | 61 |
| *Blautia luti* | 13.6 | 86.4 | 0.2 | 4.2 | 31.7 | 100.2 | 39 | 10 |
| *Blautia stercoris* | 39.5 | 60.5 | 0.7 | 0.2 | 97.6 | 178.3 | 366 | 91 |
| *Blautia wexlerae* | 24.0 | 76.0 | 0.3 | 6.4 | 29.4 | 62.6 | 33 | 8 |
| *Catenibacteriummitsuokai* | 45.7 | 54.3 | 0.8 | 0.2 | 149.9 | 284.1 | 863 | 216 |
| *Clostridium bartlettii* | 94.7 | 5.3 | 18.0 | 0.2 | 104.7 | 163.1 | 421 | 105 |
| *Clostridium celerecrescens* | 58.5 | 41.5 | 1.4 | 0.2 | 138.3 | 398.0 | 735 | 184 |
| *Clostridium disporicum* | 100.0 | 0.0 | ∞ | 0.1 | 122.0 | 188.7 | 572 | 143 |
| *Clostridium leptum* | 100.0 | 0.0 | ∞ | 0.1 | 115.7 | 178.0 | 514 | 129 |
| *Clostridium lituseburense* | 85.8 | 14.2 | 6.0 | 0.2 | 88.3 | 147.3 | 300 | 75 |
| *Clostridium xylanolyticum* | 66.2 | 33.8 | 2.0 | 0.1 | 75.6 | 252.1 | 219 | 55 |
| *Coprococcus catus* | 22.1 | 77.9 | 0.3 | 0.3 | 82.5 | 105.4 | 261 | 65 |
| *Coprococcus comes* | 4.9 | 95.1 | 0.1 | 0.7 | 60.1 | 115.1 | 139 | 35 |
| *Coprococcus eutactus* | 77.9 | 22.1 | 3.5 | 0.3 | 122.4 | 191.7 | 576 | 144 |
| *Dialistersuccinatiphilus* | 32.5 | 67.5 | 0.5 | 0.1 | 95.7 | 331.3 | 352 | 88 |
| *Dorea formicigenerans* | 54.6 | 45.4 | 1.2 | 0.2 | 81.9 | 102.3 | 258 | 64 |
| *Dorea longicatena* | 23.4 | 76.6 | 0.3 | 1.2 | 50.9 | 77.9 | 100 | 25 |
| *Eubacterium coprostanoligenes* | 22.6 | 77.4 | 0.3 | 0.1 | 117.7 | 200.8 | 532 | 133 |
| *Eubacterium desmolans* | 26.8 | 73.2 | 0.4 | 0.1 | 96.0 | 199.2 | 354 | 89 |
| *Eubacterium eligens* | 55.7 | 44.3 | 1.3 | 0.2 | 120.4 | 227.8 | 557 | 139 |
| *Eubacterium hadrum* | 9.7 | 90.3 | 0.1 | 2.0 | 60.2 | 119.2 | 139 | 35 |
| *Eubacterium hallii* | 44.5 | 55.5 | 0.8 | 1.6 | 36.9 | 53.4 | 52 | 13 |
| *Eubacterium ramulus* | 26.1 | 73.9 | 0.4 | 0.2 | 82.5 | 115.3 | 261 | 65 |
| *Eubacterium rectale* | 37.0 | 63.0 | 0.6 | 0.8 | 89.9 | 187.6 | 311 | 78 |
| *Eubacterium ruminantium* | 28.4 | 71.6 | 0.4 | 0.3 | 120.5 | 186.7 | 557 | 139 |
| *Eubacterium siraeum* | 29.1 | 70.9 | 0.4 | 0.5 | 115.0 | 181.9 | 508 | 127 |
| *Eubacterium ventriosum* | 68.5 | 31.5 | 2.2 | 0.3 | 66.4 | 99.8 | 170 | 42 |
| *Faecalibacterium prausnitzii* | 57.8 | 42.2 | 1.4 | 5.2 | 41.6 | 59.6 | 67 | 17 |
| *Lachnospirapectinoschiza* | 43.2 | 56.8 | 0.8 | 0.1 | 89.2 | 292.2 | 306 | 76 |

to be continued

**Table S3** continued

| Phylum  *Species* | Percentage contributions of variance components^a^ | | A/B | Mean^b^ | CV_w_ | CV_b_ | Days^c^ | |
| --- | --- | --- | --- | --- | --- | --- | --- | --- |
|  | Intra-individual　(A) | Inter-individual　(B) |  | (%) | (%) | (%) | 10% | 20% |
| *Megamonas funiformis* | 28.8 | 71.2 | 0.4 | 2.7 | 106.5 | 250.7 | 436 | 109 |
| *Megamonas rupellensis* | 46.2 | 53.8 | 0.9 | 0.0 | 120.8 | 311.5 | 561 | 140 |
| *Megasphaeraelsdenii* | 64.2 | 35.8 | 1.8 | 0.6 | 129.1 | 275.1 | 640 | 160 |
| *Mitsuokellamultacida* | 78.3 | 21.7 | 3.6 | 0.1 | 101.2 | 289.7 | 394 | 98 |
| *Phascolarctobacteriumfaecium* | 6.7 | 93.3 | 0.1 | 0.4 | 103.3 | 187.2 | 410 | 102 |
| *Phascolarctobacteriumsuccinatutens* | 22.5 | 77.5 | 0.3 | 0.3 | 115.8 | 189.1 | 515 | 129 |
| *Roseburia faecis* | 62.9 | 37.1 | 1.7 | 0.4 | 96.7 | 150.1 | 359 | 90 |
| *Roseburia intestinalis* | 63.7 | 36.3 | 1.8 | 0.3 | 128.2 | 249.3 | 631 | 158 |
| *Roseburia inulinivorans* | 61.6 | 38.4 | 1.6 | 0.2 | 86.0 | 136.5 | 284 | 71 |
| *Ruminococcus bromii* | 32.0 | 68.0 | 0.5 | 1.9 | 78.8 | 116.0 | 239 | 60 |
| *Ruminococcus callidus* | 56.4 | 43.6 | 1.3 | 0.4 | 84.2 | 150.4 | 272 | 68 |
| *Ruminococcus faecis* | 19.7 | 80.3 | 0.2 | 0.8 | 69.6 | 143.0 | 186 | 47 |
| *Ruminococcus gnavus* | 66.0 | 34.0 | 1.9 | 1.1 | 91.7 | 199.4 | 323 | 81 |
| *Ruminococcus lactaris* | 30.3 | 69.7 | 0.4 | 0.3 | 73.2 | 124.7 | 206 | 52 |
| *Ruminococcus obeum* | 38.7 | 61.3 | 0.6 | 0.7 | 51.7 | 93.5 | 103 | 26 |
| *Ruminococcus torques* | 79.2 | 20.8 | 3.8 | 0.7 | 85.8 | 163.1 | 283 | 71 |
| *Streptococcus salivarius* | 92.3 | 7.7 | 12.0 | 0.3 | 72.3 | 100.6 | 201 | 50 |
| *Streptococcus thermophilus* | 92.0 | 8.0 | 11.4 | 0.4 | 103.2 | 155.0 | 409 | 102 |
| *Subdoligranulum variabile* | 26.3 | 73.7 | 0.4 | 1.9 | 63.2 | 89.2 | 153 | 38 |
| *Veillonellaratti* | 19.0 | 81.0 | 0.2 | 0.4 | 148.6 | 317.0 | 848 | 212 |
| Proteobacteria |  |  |  |  |  |  |  |  |
| *Parasutterellaexcrementihominis* | 16.3 | 83.7 | 0.2 | 0.2 | 112.6 | 236.2 | 487 | 122 |
| *Sphingomonasleidyi* | 89.5 | 10.5 | 8.5 | 0.2 | 82.4 | 272.4 | 261 | 65 |
| *Sutterellastercoricanis* | 6.0 | 94.0 | 0.1 | 0.2 | 107.2 | 200.5 | 442 | 110 |
| *Sutterellawadsworthensis* | 11.6 | 88.4 | 0.1 | 0.1 | 119.7 | 206.7 | 550 | 138 |
| Verrucomicrobia |  |  |  |  |  |  |  |  |
| *Akkermansiamuciniphila* | 60.4 | 39.6 | 1.5 | 0.3 | 127.7 | 214.1 | 626 | 157 |
| Others | 16.0 | 84.0 | 0.2 | 34.1 | 10.5 | 37.0 | 4 | 1 |

^a^The inter-individual (*A*) variation represents variation between individual subjects and, intra-individual (*B*) variation represents day and residual variations.

^b^Composition mean (%) among the 10 subjects (all 40 samples).

^c^The number of days of fecal sample collection required to estimate the intake values within 10 and 20 % of their true mean with 95 % confidence.
