# Supplementary material for: Inter- and intra-individual variations in seasonal and daily stabilities of the human gut microbiota in Japanese
Source: Arch Microbiol. 2015 Jun 12;197(7):919–34. doi: 10.1007/s00203-015-1125-0 (PMC4536265; doi:10.1007/s00203-015-1125-0)
Supplement: Supplementary file 4 — Supplementary material 4 (DOCX 38 kb) [file 203_2015_1125_MOESM4_ESM.docx]

**TableS4**Relative contributions of intra- and inter-individual variance in 83 selected dominant species, coefficient of within-person variance (CV_w_) and between-person variance (CV_b_), and the number of days ("Days") of fecal sample collection required to estimate the true values within 10 and 20 % of their true mean, based on daily stability

| Phylum  *Species* | Percentage contributions of variance components^a^ | | *A*/*B* | Mean^b^ | CV_w_ | CV_b_ | Days^ｃ^ | |
| --- | --- | --- | --- | --- | --- | --- | --- | --- |
|  | Intra-individual　(*A*) | Inter-individual　(*B*) |  | (%) | (%) | (%) | 10% | 20% |
| Actinobacteria |  |  |  |  |  |  |  |  |
| *Bifidobacterium longum* | 19.9 | 80.1 | 0.2 | 1.2 | 54.1 | 128.6 | 112 | 28 |
| *Collinsellaaerofaciens* | 16.2 | 83.8 | 0.2 | 2.8 | 37.9 | 83.2 | 55 | 14 |
| *Eggerthellalenta* | 41.9 | 58.1 | 0.7 | 0.1 | 42.7 | 112.6 | 70 | 18 |
| Bacteroidetes |  |  |  |  |  |  |  |  |
| *Alistipesonderdonkii* | 18.1 | 81.9 | 0.2 | 0.3 | 104.9 | 203.2 | 423 | 106 |
| *Alistipesputredinis* | 30.0 | 70.0 | 0.4 | 0.2 | 98.7 | 143.1 | 374 | 94 |
| *Alistipesshahii* | 17.6 | 82.4 | 0.2 | 0.2 | 107.7 | 171.6 | 446 | 111 |
| *Bacteroides clarus* | 20.1 | 79.9 | 0.3 | 0.0 | 119.0 | 234.8 | 544 | 136 |
| *Bacteroides coprocola* | 32.3 | 67.7 | 0.5 | 0.4 | 110.4 | 290.2 | 468 | 117 |
| *Bacteroides dorei* | 16.8 | 83.2 | 0.2 | 1.3 | 59.1 | 142.3 | 134 | 34 |
| *Bacteroides eggerthii* | 35.1 | 64.9 | 0.5 | 0.1 | 140.5 | 307.9 | 758 | 189 |
| *Bacteroides faecichinchillae* | 13.9 | 86.1 | 0.2 | 0.2 | 102.0 | 165.8 | 400 | 100 |
| *Bacteroides faecis* | 25.5 | 74.5 | 0.3 | 0.3 | 103.7 | 201.2 | 413 | 103 |
| *Bacteroides finegoldii* | 16.7 | 83.3 | 0.2 | 0.2 | 115.8 | 231.8 | 515 | 129 |
| *Bacteroides fragilis* | 53.3 | 46.7 | 1.1 | 0.2 | 116.6 | 211.6 | 522 | 131 |
| *Bacteroides massiliensis* | 13.3 | 86.7 | 0.2 | 0.5 | 153.7 | 245.1 | 907 | 227 |
| *Bacteroides ovatus* | 7.7 | 92.3 | 0.1 | 0.9 | 73.3 | 206.6 | 206 | 52 |
| *Bacteroides plebeius* | 20.3 | 79.7 | 0.3 | 2.7 | 74.7 | 176.9 | 214 | 54 |
| *Bacteroides stercoris* | 9.3 | 90.7 | 0.1 | 0.1 | 110.9 | 290.7 | 472 | 118 |
| *Bacteroides uniformis* | 13.9 | 86.1 | 0.2 | 2.0 | 64.4 | 144.6 | 159 | 40 |
| *Bacteroides vulgatus* | 14.4 | 85.6 | 0.2 | 4.1 | 62.1 | 105.7 | 148 | 37 |
| *Bacteroides xylanisolvens* | 23.7 | 76.3 | 0.3 | 0.5 | 55.3 | 121.6 | 117 | 29 |
| *Barnesiellaintestinihominis* | 15.8 | 84.2 | 0.2 | 0.1 | 140.0 | 206.5 | 753 | 188 |
| *Odoribactersplanchnicus* | 23.2 | 76.8 | 0.3 | 0.1 | 103.8 | 145.6 | 414 | 103 |
| *Parabacteroides distasonis* | 22.4 | 77.6 | 0.3 | 0.6 | 76.9 | 123.1 | 227 | 57 |
| *Parabacteroides johnsonii* | 30.5 | 69.5 | 0.4 | 0.1 | 126.5 | 308.5 | 615 | 154 |
| *Parabacteroides merdae* | 30.5 | 69.5 | 0.4 | 0.2 | 117.6 | 209.2 | 532 | 133 |
| *Prevotella copri* | 29.0 | 71.0 | 0.4 | 2.0 | 92.0 | 198.3 | 325 | 81 |
| *Prevotella stercorea* | 43.3 | 56.7 | 0.8 | 0.2 | 150.8 | 310.6 | 874 | 218 |

to be continued

**Table S4**continued

| Phylum  *Species* | Percentage contributions of variance components^a^ | | *A*/*B* | Mean^b^ | CV_w_ | CV_b_ | Days^ｃ^ | |
| --- | --- | --- | --- | --- | --- | --- | --- | --- |
|  | Intra-individual　(*A*) | Inter-individual　(*B*) |  | (%) | (%) | (%) | 10% | 20% |
| Firmicutes |  |  |  |  |  |  |  |  |
| *Blautia faecis* | 13.6 | 86.4 | 0.2 | 0.8 | 36.4 | 96.7 | 51 | 13 |
| *Blautia glucerasea* | 20.6 | 79.4 | 0.3 | 0.1 | 56.8 | 128.5 | 124 | 31 |
| *Blautia luti* | 8.5 | 91.5 | 0.1 | 4.3 | 28.8 | 91.5 | 32 | 8 |
| *Blautia stercoris* | 19.2 | 80.8 | 0.2 | 0.2 | 99.4 | 187.4 | 379 | 95 |
| *Blautia wexlerae* | 15.8 | 84.2 | 0.2 | 7.1 | 24.0 | 56.0 | 22 | 6 |
| *Catenibacteriummitsuokai* | 15.3 | 84.7 | 0.2 | 0.1 | 142.9 | 216.7 | 785 | 196 |
| *Clostridium bartlettii* | 54.7 | 45.3 | 1.2 | 0.2 | 79.3 | 108.5 | 242 | 60 |
| *Clostridium celerecrescens* | 37.7 | 62.3 | 0.6 | 0.1 | 149.1 | 308.8 | 854 | 214 |
| *Clostridium disporicum* | 33.9 | 66.1 | 0.5 | 0.2 | 102.9 | 167.7 | 407 | 102 |
| *Clostridium leptum* | 25.1 | 74.9 | 0.3 | 0.1 | 114.8 | 280.8 | 506 | 127 |
| *Clostridium lituseburense* | 57.0 | 43.0 | 1.3 | 0.2 | 62.3 | 136.9 | 149 | 37 |
| *Clostridium xylanolyticum* | 10.6 | 89.4 | 0.1 | 0.2 | 72.2 | 197.7 | 200 | 50 |
| *Coprococcus catus* | 18.0 | 82.0 | 0.2 | 0.3 | 79.3 | 102.8 | 241 | 60 |
| *Coprococcus comes* | 4.0 | 96.0 | 0.0 | 0.5 | 59.3 | 102.3 | 135 | 34 |
| *Coprococcus eutactus* | 78.8 | 21.2 | 3.7 | 0.2 | 127.4 | 220.6 | 624 | 156 |
| *Dialistersuccinatiphilus* | 28.6 | 71.4 | 0.4 | 0.1 | 121.3 | 300.8 | 566 | 141 |
| *Dorea formicigenerans* | 12.5 | 87.5 | 0.1 | 0.2 | 61.7 | 89.6 | 146 | 37 |
| *Dorea longicatena* | 8.2 | 91.8 | 0.1 | 1.2 | 30.8 | 66.7 | 36 | 9 |
| *Eubacterium coprostanoligenes* | 39.1 | 60.9 | 0.6 | 0.1 | 115.7 | 175.3 | 515 | 129 |
| *Eubacterium desmolans* | 10.9 | 89.1 | 0.1 | 0.1 | 99.7 | 215.0 | 382 | 95 |
| *Eubacterium eligens* | 11.4 | 88.6 | 0.1 | 0.4 | 129.3 | 192.1 | 642 | 161 |
| *Eubacterium hadrum* | 17.9 | 82.1 | 0.2 | 2.5 | 47.9 | 96.9 | 88 | 22 |
| *Eubacterium hallii* | 41.0 | 59.0 | 0.7 | 1.5 | 36.6 | 60.7 | 51 | 13 |
| *Eubacterium ramulus* | 12.5 | 87.5 | 0.1 | 0.1 | 66.0 | 121.6 | 168 | 42 |
| *Eubacterium rectale* | 15.3 | 84.7 | 0.2 | 0.5 | 80.3 | 145.0 | 248 | 62 |
| *Eubacterium ruminantium* | 11.7 | 88.3 | 0.1 | 0.2 | 161.3 | 279.8 | 1000 | 250 |
| *Eubacterium siraeum* | 42.3 | 57.7 | 0.7 | 0.3 | 142.8 | 215.7 | 783 | 196 |
| *Eubacterium ventriosum* | 17.6 | 82.4 | 0.2 | 0.3 | 58.6 | 100.8 | 132 | 33 |
| *Faecalibacterium prausnitzii* | 37.7 | 62.3 | 0.6 | 6.9 | 34.3 | 51.6 | 45 | 11 |
| *Lachnospirapectinoschiza* | 7.1 | 92.9 | 0.1 | 0.3 | 105.2 | 283.6 | 425 | 106 |
| *Megamonas funiformis* | 26.6 | 73.4 | 0.4 | 2.5 | 95.8 | 239.8 | 353 | 88 |

to be continued

**Table S4**continued

| Phylum  *Species* | Percentage contributions of variance components^a^ | | A/B | Mean^b^ | CV_w_ | CV_b_ | Days^ｃ^ | |
| --- | --- | --- | --- | --- | --- | --- | --- | --- |
|  | Intra-individual　(A) | Inter-individual　(B) |  | (%) | (%) | (%) | 10% | 20% |
| *Megamonas funiformis* | 26.6 | 73.4 | 0.4 | 2.5 | 95.8 | 239.8 | 353 | 88 |
| *Megamonas rupellensis* | 66.7 | 33.3 | 2.0 | 0.1 | 141.4 | 322.3 | 768 | 192 |
| *Megasphaeraelsdenii* | 43.3 | 56.7 | 0.8 | 0.7 | 145.5 | 258.2 | 813 | 203 |
| *Mitsuokellamultacida* | 56.4 | 43.6 | 1.3 | 0.4 | 170.9 | 320.9 | 1122 | 281 |
| *Phascolarctobacteriumfaecium* | 8.6 | 91.4 | 0.1 | 0.3 | 99.3 | 170.6 | 379 | 95 |
| *Phascolarctobacteriumsuccinatutens* | 19.8 | 80.2 | 0.2 | 0.2 | 134.7 | 177.1 | 697 | 174 |
| *Roseburia faecis* | 17.0 | 83.0 | 0.2 | 0.7 | 68.5 | 128.1 | 180 | 45 |
| *Roseburia intestinalis* | 29.3 | 70.7 | 0.4 | 0.3 | 129.2 | 194.0 | 642 | 160 |
| *Roseburia inulinivorans* | 24.6 | 75.4 | 0.3 | 0.3 | 69.0 | 124.8 | 183 | 46 |
| *Ruminococcus bromii* | 6.6 | 93.4 | 0.1 | 1.1 | 85.5 | 171.7 | 281 | 70 |
| *Ruminococcus callidus* | 23.1 | 76.9 | 0.3 | 0.3 | 137.1 | 166.6 | 722 | 180 |
| *Ruminococcus faecis* | 6.2 | 93.8 | 0.1 | 0.8 | 70.2 | 156.3 | 190 | 47 |
| *Ruminococcus gnavus* | 22.2 | 77.8 | 0.3 | 0.8 | 54.5 | 129.2 | 114 | 29 |
| *Ruminococcus lactaris* | 12.5 | 87.5 | 0.1 | 0.4 | 68.3 | 122.8 | 179 | 45 |
| *Ruminococcus obeum* | 21.3 | 78.7 | 0.3 | 0.7 | 45.0 | 86.9 | 78 | 19 |
| *Ruminococcus torques* | 52.6 | 47.4 | 1.1 | 0.4 | 75.0 | 124.1 | 216 | 54 |
| *Streptococcus salivarius* | 17.2 | 82.8 | 0.2 | 0.4 | 39.5 | 109.1 | 60 | 15 |
| *Streptococcus thermophilus* | 52.0 | 48.0 | 1.1 | 0.5 | 94.7 | 194.8 | 344 | 86 |
| *Subdoligranulum variabile* | 17.0 | 83.0 | 0.2 | 1.9 | 41.0 | 92.2 | 65 | 16 |
| *Veillonellaratti* | 18.4 | 81.6 | 0.2 | 0.4 | 151.3 | 289.7 | 880 | 220 |
| Proteobacteria |  |  |  |  |  |  |  |  |
| *Parasutterellaexcrementihominis* | 2.3 | 97.7 | 0.0 | 0.2 | 141.5 | 246.0 | 769 | 192 |
| *Sphingomonasleidyi* | 71.7 | 28.3 | 2.5 | 0.9 | 62.1 | 111.3 | 148 | 37 |
| *Sutterellastercoricanis* | 9.7 | 90.3 | 0.1 | 0.2 | 139.3 | 193.8 | 745 | 186 |
| *Sutterellawadsworthensis* | 14.9 | 85.1 | 0.2 | 0.1 | 95.2 | 290.5 | 348 | 87 |
| Verrucomicrobia |  |  |  |  |  |  |  |  |
| *Akkermansiamuciniphila* | 23.7 | 76.3 | 0.3 | 0.6 | 121.3 | 387.8 | 565 | 141 |
| Others | 31.7 | 68.3 | 0.5 | 33.7 | 15.9 | 32.0 | 10 | 2 |

^a^The inter-individual (*A*) variation represents variation between individual subjects and, intra-individual (*B*) variation represents day and residual variations.

^b^Composition mean (%) among the 10 subjects (all 72 samples).

^c^The number of days of fecal sample collection required to estimate the intake values within 10 and 20 % of their true mean with 95 % confidence.
