# Supplementary material for: Inter- and intra-individual variations in seasonal and daily stabilities of the human gut microbiota in Japanese
Source: Arch Microbiol. 2015 Jun 12;197(7):919–34. doi: 10.1007/s00203-015-1125-0 (PMC4536265; doi:10.1007/s00203-015-1125-0)
Supplement: Supplementary file 5 — Supplementary material 5 (DOCX 50 kb) [file 203_2015_1125_MOESM5_ESM.docx]

**Table A1** Compositions (%) of 39 selected dominant genera in the human gut microbiota of the 10 subjects (S1–S10), based on seasonal stability

| Phylum | Subject (S1–S10) | | | | | | | | | |  | *P* value^b^ | |
| --- | --- | --- | --- | --- | --- | --- | --- | --- | --- | --- | --- | --- | --- |
| *Genus* | S1 | S2 | S3 | S4 | S5 | S6 | S7 | S8 | S9 | S10 |  | Subject | Season |
| Actinobacteria |  |  |  |  |  |  |  |  |  |  |  |  |  |
| *Bifidobacterium* | 4.0 ± 4.2^a^ | 8.6 ± 6.9 | 8.8 ± 3.6 | 1.6 ± 1.3 | 2.0 ± 2.8 | 6.2 ± 5.8 | 0.3 ± 0.5 | 4.5 ± 4.7 | 4.1 ± 3.2 | 6.2 ± 4.5 |  | 0.033 | 0.241 |
| *Collinsella* | 5.1 ± 1.6 | 4.7 ± 1.1 | 0.1 ± 0.1 | 0.4 ± 0.6 | 3.8 ± 0.7 | 3.7 ± 0.2 | 1.5 ± 0.9 | 3.5 ± 1.3 | 1.5 ± 0.3 | 2.8 ± 1.1 |  | 0.001 | 0.167 |
| *Eggerthella* | 0.1 ± 0.1 | 0.0 ± 0.0 | 0.2 ± 0.1 | 0.0 ± 0.0 | 0.0 ± 0.0 | 0.0 ± 0.0 | 0.3 ± 0.1 | 0.1 ± 0.1 | −^c^ | 0.1 ± 0.0 |  | 0.008 | 0.850 |
| Bacteroidetes |  |  |  |  |  |  |  |  |  |  |  |  |  |
| *Alistipes* | 1.2 ± 0.5 | 0.9 ± 0.7 | 0.5 ± 0.3 | 2.2 ± 0.5 | 1.2 ± 0.3 | 2.4 ± 0.8 | 0.0 ± 0.0 | 0.0 ± 0.0 | 0.2 ± 0.1 | 0.7 ± 0.4 |  | <0.001 | 0.416 |
| *Bacteroides* | 11.4 ± 1.3 | 24.0 ± 7.2 | 25.6 ± 4.6 | 10.4 ± 4.6 | 17.5 ± 3.3 | 9.7 ± 3.5 | 34.3 ± 3.6 | 20.0 ± 5.7 | 1.2 ± 0.7 | 22.5 ± 4.7 |  | 0.001 | 0.241 |
| *Barnesiella* | 0.4 ± 0.2 | − | 0.0 ± 0.0 | 0.8 ± 0.4 | − | 0.1 ± 0.0 | − | − | 0.0 ± 0.0 | 0.0 ± 0.0 |  | <0.001 | 0.082 |
| *Butyricimonas* | 0.1 ± 0.0 | 0.1 ± 0.1 | 0.0 ± 0.1 | 0.1 ± 0.2 | 0.1 ± 0.1 | 0.2 ± 0.0 | 0.2 ± 0.1 | 0.0 ± 0.0 | 0.3 ± 0.1 | 0.0 ± 0.0 |  | 0.004 | 0.700 |
| *Odoribacter* | 0.5 ± 0.5 | 0.0 ± 0.0 | 0.1 ± 0.1 | 0.2 ± 0.1 | 0.2 ± 0.0 | 0.3 ± 0.0 | 0.0 ± 0.0 | 0.0 ± 0.0 | 0.0 ± 0.1 | 0.2 ± 0.1 |  | 0.001 | 0.944 |
| *Parabacteroides* | 0.8 ± 0.2 | 3.3 ± 3.6 | 1.8 ± 0.7 | 2.5 ± 1.1 | 0.0 ± 0.0 | 1.5 ± 0.3 | 0.1 ± 0.0 | 1.6 ± 0.8 | 1.1 ± 0.4 | 1.6 ± 1.6 |  | 0.006 | 0.868 |
| *Prevotella* | 10.2 ± 6.5 | 0.3 ± 0.3 | 0.0 ± 0.0 | 0.3 ± 0.4 | 0.0 ± 0.0 | 1.0 ± 1.4 | 0.0 ± 0.0 | 0.0 ± 0.0 | 2.6 ± 2.0 | 0.1 ± 0.1 |  | 0.001 | 0.025 |
| Firmicutes |  |  |  |  |  |  |  |  |  |  |  |  |  |
| *Acetivibrio* | 0.0 ± 0.0 | − | 0.0 ± 0.0 | 0.5 ± 0.3 | 0.0 ± 0.0 | 0.0 ± 0.0 | 0.0 ± 0.0 | 0.0 ± 0.0 | 0.0 ± 0.0 | − |  | 0.046 | 0.550 |
| *Anaerostipes* | 0.2 ± 0.1 | 1.6 ± 0.9 | 3.2 ± 0.5 | 1.9 ± 1.5 | 0.1 ± 0.1 | 1.2 ± 0.1 | 7.9 ± 0.6 | 0.1 ± 0.1 | 0.3 ± 0.3 | 4.6 ± 1.1 |  | <0.001 | 0.825 |
| *Bacillus* | 0.0 ± 0.0 | − | 0.0 ± 0.0 | 0.1 ± 0.1 | 0.3 ± 0.3 | 0.0 ± 0.0 | 0.3 ± 0.3 | 0.1 ± 0.2 | 0.2 ± 0.2 | 0.0 ± 0.1 |  | 0.189 | 0.263 |
| *Blautia* | 9.7 ± 3.5 | 14.5 ± 1.3 | 15.2 ± 3.3 | 14.7 ± 5.1 | 22.2 ± 3.9 | 9.8 ± 1.9 | 21.3 ± 1.4 | 17.2 ± 7.1 | 8.8 ± 3.2 | 14.7 ± 1.6 |  | 0.009 | 0.431 |
| *Catenibacterium* | 0.0 ± 0.0 | 0.0 ± 0.1 | 0.0 ± 0.0 | 1.3 ± 1.6 | − | 0.0 ± 0.0 | 0.0 ± 0.0 | 0.0 ± 0.0 | 4.9 ± 2.4 | 0.0 ± 0.0 |  | <0.001 | 0.392 |
| *Clostridium* | 2.4 ± 0.7 | 3.6 ± 0.4 | 3.7 ± 1.1 | 3.6 ± 0.8 | 1.8 ± 0.9 | 2.3 ± 0.6 | 3.7 ± 0.6 | 3.7 ± 2.7 | 2.9 ± 0.8 | 3.1 ± 0.6 |  | 0.077 | 0.786 |
| *Coprococcus* | 2.3 ± 0.5 | 0.0 ± 0.0 | 1.3 ± 0.3 | 1.4 ± 1.6 | 3.6 ± 0.3 | 1.6 ± 0.7 | 1.2 ± 0.1 | 0.0 ± 0.0 | 1.7 ± 0.6 | 0.6 ± 0.1 |  | 0.001 | 0.954 |
| *Dialister* | 0.0 ± 0.0 | 0.0 ± 0.0 | 0.0 ± 0.0 | 0.0 ± 0.0 | 0.0 ± 0.0 | 0.8 ± 0.3 | 0.0 ± 0.0 | 1.3 ± 0.7 | 0.7 ± 0.4 | 0.0 ± 0.0 |  | <0.001 | 0.265 |
| *Dorea* | 1.8 ± 0.3 | 1.5 ± 0.5 | 1.9 ± 0.3 | 0.3 ± 0.4 | 0.0 ± 0.0 | 1.0 ± 0.3 | 3.0 ± 0.8 | 2.3 ± 1.0 | 0.8 ± 0.2 | 1.5 ± 0.4 |  | 0.001 | 0.001 |
| *Eubacterium* | 5.7 ± 0.4 | 2.9 ± 0.5 | 4.8 ± 1.3 | 7.4 ± 1.5 | 5.4 ± 1.1 | 4.8 ± 0.8 | 1.8 ± 0.8 | 1.8 ± 1.0 | 5.3 ± 1.0 | 8.2 ± 2.6 |  | <0.001 | 0.321 |
| *Faecalibacterium* | 7.4 ± 2.9 | 4.7 ± 1.5 | 2.0 ± 1.2 | 7.0 ± 1.5 | 6.7 ± 2.9 | 5.2 ± 2.2 | 7.8 ± 3.7 | 1.0 ± 0.7 | 3.7 ± 1.7 | 7.5 ± 0.7 |  | 0.021 | 0.441 |
| *Lachnospira* | 0.0 ± 0.0 | 1.6 ± 0.9 | 0.2 ± 0.2 | 1.0 ± 0.6 | 0.3 ± 0.2 | 0.6 ± 0.1 | 0.0 ± 0.0 | 1.0 ± 0.7 | 0.3 ± 0.2 | 0.2 ± 0.1 |  | 0.017 | 0.340 |
| *Megamonas* | 7.2 ± 6.1 | 0.0 ± 0.0 | 0.0 ± 0.0 | 0.1 ± 0.1 | 0.0 ± 0.0 | 0.2 ± 0.2 | 0.2 ± 0.3 | 20.3 ± 8.8 | 0.1 ± 0.1 | 0.1 ± 0.1 |  | 0.002 | 0.220 |
| *Megasphaera* | 1.7 ± 1.6 | 0.0 ± 0.0 | 0.0 ± 0.0 | 0.0 ± 0.0 | − | 0.7 ± 0.4 | 0.0 ± 0.1 | 4.3 ± 3.9 | 0.0 ± 0.0 | 0.0 ± 0.0 |  | <0.001 | 0.392 |
| *Mitsuokella* | − | 0.4 ± 0.7 | − | − | − | 0.6 ± 0.4 | − | 0.0 ± 0.0 | 0.1 ± 0.1 | − |  | 0.019 | 0.218 |
| *Oscillibacter* | 0.8 ± 0.4 | 0.1 ± 0.1 | 0.2 ± 0.1 | 1.3 ± 0.5 | 1.0 ± 0.2 | 0.6 ± 0.2 | 0.0 ± 0.0 | 0.1 ± 0.0 | 1.3 ± 0.3 | 0.3 ± 0.2 |  | <0.001 | 0.724 |
| *Phascolarctobacterium* | 1.0 ± 0.4 | 1.3 ± 0.5 | 0.1 ± 0.1 | 0.7 ± 0.4 | 1.4 ± 0.3 | 0.0 ± 0.0 | 2.2 ± 0.4 | 0.0 ± 0.0 | 1.5 ± 0.3 | 1.1 ± 0.2 |  | 0.001 | 0.980 |
| *Pseudoflavonifractor* | 0.2 ± 0.1 | 0.1 ± 0.1 | 0.1 ± 0.1 | 0.2 ± 0.1 | 0.4 ± 0.0 | 0.5 ± 0.1 | 0.0 ± 0.0 | 0.2 ± 0.2 | 0.3 ± 0.1 | 0.1 ± 0.1 |  | 0.002 | 0.220 |
| *Roseburia* | 0.4 ± 0.3 | 3.7 ± 0.9 | 0.3 ± 0.4 | 1.6 ± 0.4 | 0.7 ± 0.7 | 1.0 ± 0.4 | 0.4 ± 0.2 | 0.2 ± 0.2 | 0.8 ± 0.7 | 0.8 ± 0.3 |  | 0.007 | 0.455 |
| *Ruminococcus* | 7.1 ± 1.1 | 6.5 ± 1.9 | 3.2 ± 1.7 | 7.7 ± 0.4 | 9.2 ± 3.0 | 10.1 ± 0.7 | 8.1 ± 1.0 | 1.6 ± 1.0 | 7.3 ± 2.7 | 10.5 ± 0.9 |  | 0.002 | 0.172 |
| *Sporobacter* | 0.1 ± 0.1 | 0.0 ± 0.0 | 0.0 ± 0.0 | 0.2 ± 0.0 | 0.2 ± 0.1 | 0.4 ± 0.1 | − | 0.0 ± 0.0 | 1.0 ± 0.5 | 0.0 ± 0.0 |  | <0.001 | 0.360 |
| *Streptococcus* | 1.3 ± 0.8 | 1.8 ± 1.1 | 10.4 ± 2.6 | 2.0 ± 1.3 | 0.7 ± 0.5 | 0.3 ± 0.2 | 1.0 ± 0.3 | 6.4 ± 3.2 | 0.5 ± 0.4 | 0.7 ± 0.4 |  | 0.003 | 0.700 |
| *Subdoligranulum* | 2.8 ± 1.1 | 2.1 ± 1.0 | 3.3 ± 1.8 | 1.0 ± 1.6 | 10.0 ± 1.8 | 2.2 ± 0.6 | 0.1 ± 0.1 | 0.6 ± 0.5 | 6.6 ± 1.5 | 2.1 ± 0.7 |  | 0.001 | 0.469 |
| *Veillonella* | 0.1 ± 0.1 | 0.2 ± 0.3 | 4.5 ± 1.8 | 0.1 ± 0.2 | 0.0 ± 0.0 | 0.0 ± 0.0 | 0.1 ± 0.0 | 0.1 ± 0.1 | 0.0 ± 0.0 | 0.0 ± 0.0 |  | 0.013 | 0.954 |
| Proteobacteria |  |  |  |  |  |  |  |  |  |  |  |  |  |
| *Brevundimonas* | 0.1 ± 0.1 | 0.1 ± 0.1 | 0.0 ± 0.0 | 0.2 ± 0.4 | 0.0 ± 0.0 | 0.1 ± 0.1 | 0.0 ± 0.0 | 0.0 ± 0.0 | 0.9 ± 1.2 | 0.1 ± 0.0 |  | 0.377 | 0.601 |
| *Mesorhizobium* | 0.0 ± 0.0 | 0.0 ± 0.0 | 0.0 ± 0.0 | 0.1 ± 0.1 | − | 0.0 ± 0.0 | 0.0 ± 0.0 | 0.0 ± 0.0 | 0.1 ± 0.1 | 0.0 ± 0.0 |  | 0.135 | 0.706 |
| *Parasutterella* | 0.0 ± 0.0 | 1.5 ± 0.5 | 0.0 ± 0.0 | 0.1 ± 0.1 | 0.5 ± 0.1 | 0.4 ± 0.1 | 0.0 ± 0.0 | 0.0 ± 0.0 | 0.0 ± 0.0 | 0.0 ± 0.0 |  | <0.001 | 0.589 |
| *Sutterella* | 1.0 ± 0.2 | 0.0 ± 0.0 | 0.0 ± 0.0 | 0.1 ± 0.3 | 0.8 ± 0.2 | 0.0 ± 0.0 | 0.0 ± 0.0 | 0.0 ± 0.0 | 0.1 ± 0.1 | 1.4 ± 0.2 |  | <0.001 | 0.123 |
| Verrucomicrobia |  |  |  |  |  |  |  |  |  |  |  |  |  |
| *Akkermansia* | 0.0 ± 0.1 | 0.0 ± 0.0 | 0.1 ± 0.2 | 1.1 ± 0.5 | 0.0 ± 0.0 | 0.3 ± 0.4 | 0.0 ± 0.0 | 0.0 ± 0.0 | 0.0 ± 0.0 | 1.1 ± 1.0 |  | 0.010 | 0.875 |
| Others | 12.9 ± 2.9 | 9.7 ± 0.5 | 8.2 ± 1.2 | 25.7 ± 3.8 | 9.8 ± 2.4 | 30.2 ± 4.0 | 4.0 ± 0.4 | 7.9 ± 3.5 | 38.9 ± 7.9 | 6.9 ± 1.7 |  | <0.001 | 0.564 |

^a^ The individual means ± SD was calculated using all values for 1 year.

^b^ Based on Friedman test, the two variables “Subject” and “Season”, correspond to “inter-” and “intra-” individual variations, respectively.

^C^ Not detected.
