# Supplementary material for: Inter- and intra-individual variations in seasonal and daily stabilities of the human gut microbiota in Japanese
Source: Arch Microbiol. 2015 Jun 12;197(7):919–34. doi: 10.1007/s00203-015-1125-0 (PMC4536265; doi:10.1007/s00203-015-1125-0)
Supplement: Supplementary file 6 — Supplementary material 6 (DOCX 50 kb) [file 203_2015_1125_MOESM6_ESM.docx]

**Table A2** Compositions (%) of 39 selected dominant genera in the human gut microbiota for the 10 subjects (S1–S10), based on daily stability

| Phylum | Subject (S1–S10) | | | | | | | | | |  | *P* value^b^ | |
| --- | --- | --- | --- | --- | --- | --- | --- | --- | --- | --- | --- | --- | --- |
| *Genus* | S1 | S2 | S3 | S4 | S5 | S6 | S7 | S8 | S9 | S10 |  | Subject | Day |
| Actinobacteria |  |  |  |  |  |  |  |  |  |  |  |  |  |
| *Bifidobacterium* | 5.3 ± 2.2^a^ | 11.0 ± 4.4 | 4.4 ± 1.7 | 0.8 ± 0.2 | 5.9 ± 1.0 | 1.7 ± 0.8 | 0.1 ± 0.1 | 3.4 ± 1.2 | 4.6 ± 2.7 | 5.0 ± 1.0 |  | <0.001 | 0.427 |
| *Collinsella* | 5.1 ± 1.2 | 6.0 ± 1.1 | 0.1 ± 0.1 | 0.0 ± 0.0 | 3.2 ± 0.4 | 4.6 ± 0.4 | 1.1 ± 0.3 | 5.0 ± 1.9 | 3.4 ± 1.4 | 3.2 ± 0.7 |  | <0.001 | 0.916 |
| *Eggerthella* | 0.0 ± 0.0 | 0.1 ± 0.0 | 0.1 ± 0.1 | 0.1 ± 0.0 | 0.1 ± 0.1 | 0.0 ± 0.0 | 0.3 ± 0.1 | 0.3 ± 0.2 | −^ｃ^ | 0.1 ± 0.0 |  | <0.001 | 0.119 |
| Bacteroidetes |  |  |  |  |  |  |  |  |  |  |  |  |  |
| *Alistipes* | 0.4 ± 0.4 | 0.7 ± 0.4 | 0.1 ± 0.1 | 1.9 ± 0.6 | 1.3 ± 0.2 | 2.7 ± 1.1 | 0.1 ± 0.1 | 0.4 ± 0.9 | 0.1 ± 0.1 | 0.8 ± 0.4 |  | <0.001 | 0.631 |
| *Bacteroides* | 8.9 ± 4.7 | 17.6 ± 3.9 | 18.5 ± 6.8 | 6.4 ± 2.0 | 10.1 ± 2.0 | 9.2 ± 3.5 | 27.9 ± 4.7 | 20.1 ± 2.5 | 0.6 ± 0.3 | 14.6 ± 1.9 |  | <0.001 | 0.714 |
| *Barnesiella* | 0.3 ± 0.3 | 0.0 ± 0.0 | 0.0 ± 0.0 | 0.7 ± 0.1 | 0.0 ± 0.0 | 0.1 ± 0.0 | 0.0 ± 0.0 | 0.1 ± 0.1 | 0.0 ± 0.0 | 0.0 ± 0.0 |  | <0.001 | 0.614 |
| *Butyricimonas* | 0.0 ± 0.0 | 0.0 ± 0.0 | 0.0 ± 0.0 | − | 0.0 ± 0.0 | 0.0 ± 0.0 | 0.4 ± 0.1 | 0.0 ± 0.0 | 0.0 ± 0.0 | 0.0 ± 0.0 |  | <0.001 | 0.423 |
| *Odoribacter* | 0.1 ± 0.2 | − | 0.0 ± 0.0 | 0.2 ± 0.1 | 0.2 ± 0.0 | 0.3 ± 0.0 | 0.0 ± 0.0 | 0.1 ± 0.1 | 0.0 ± 0.0 | 0.2 ± 0.1 |  | <0.001 | 0.974 |
| *Parabacteroides* | 0.5 ± 0.3 | 1.3 ± 0.8 | 0.7 ± 0.4 | 2.8 ± 0.7 | 0.0 ± 0.0 | 1.4 ± 0.9 | 0.0 ± 0.0 | 1.7 ± 0.6 | 0.4 ± 0.2 | 1.2 ± 0.7 |  | <0.001 | 0.357 |
| *Prevotella* | 17.4 ± 6.5 | 0.0 ± 0.0 | 0.5 ± 0.5 | 0.1 ± 0.0 | 0.0 ± 0.0 | 2.4 ± 2.7 | 0.5 ± 0.2 | 0.1 ± 0.1 | 15.9 ± 10.9 | 0.1 ± 0.1 |  | <0.001 | 0.348 |
| Firmicutes |  |  |  |  |  |  |  |  |  |  |  |  |  |
| *Acetivibrio* | 0.0 ± 0.0 | 0.0 ± 0.0 | 0.0 ± 0.0 | 2.0 ± 0.3 | 0.0 ± 0.0 | 0.0 ± 0.0 | − | 0.1 ± 0.2 | 0.0 ± 0.0 | 0.0 ± 0.0 |  | <0.001 | 0.477 |
| *Anaerostipes* | 0.3 ± 0.1 | 2.2 ± 0.7 | 5.2 ± 2.7 | 3.2 ± 0.5 | 0.1 ± 0.0 | 1.1 ± 0.3 | 6.4 ± 0.8 | 0.3 ± 0.4 | 0.4 ± 0.1 | 4.3 ± 0.8 |  | <0.001 | 0.541 |
| *Bacillus* | 0.1 ± 0.1 | 0.0 ± 0.0 | 0.0 ± 0.0 | 0.1 ± 0.0 | 0.2 ± 0.2 | 0.0 ± 0.0 | 0.0 ± 0.0 | 0.0 ± 0.0 | 0.1 ± 0.1 | 0.0 ± 0.0 |  | <0.001 | 0.486 |
| *Blautia* | 6.9 ± 4.5 | 16.3 ± 1.5 | 20.8 ± 2.3 | 17.3 ± 0.9 | 16.2 ± 1.6 | 7.9 ± 0.7 | 18.9 ± 2.8 | 16.7 ± 5.8 | 7.5 ± 2.1 | 16.1 ± 1.2 |  | <0.001 | 0.563 |
| *Catenibacterium* | 0.0 ± 0.0 | 0.0 ± 0.0 | 0.0 ± 0.0 | 0.4 ± 0.1 | − | 0.0 ± 0.0 | 0.0 ± 0.0 | 0.1 ± 0.1 | 2.4 ± 0.8 | 0.0 ± 0.0 |  | <0.001 | 0.471 |
| *Clostridium* | 3.3 ± 1.0 | 3.7 ± 0.3 | 6.9 ± 1.8 | 3.9 ± 0.6 | 2.1 ± 0.5 | 2.4 ± 0.3 | 5.7 ± 0.8 | 2.3 ± 0.4 | 2.0 ± 0.5 | 3.4 ± 0.5 |  | <0.001 | 0.848 |
| *Coprococcus* | 1.5 ± 0.8 | 0.0 ± 0.0 | 1.0 ± 0.2 | 0.5 ± 0.1 | 2.8 ± 0.1 | 1.3 ± 0.1 | 1.0 ± 0.2 | 0.7 ± 1.4 | 2.0± 0.5 | 0.7 ± 0.1 |  | <0.001 | 0.330 |
| *Dialister* | − | 0.0 ± 0.0 | 0.0 ± 0.0 | 0.0 ± 0.0 | 0.0 ± 0.0 | 1.3 ± 0.5 | 0.1 ± 0.0 | 1.0 ± 0.5 | 2.1 ± 0.4 | 0.0 ± 0.0 |  | <0.001 | 0.956 |
| *Dorea* | 1.6 ± 0.4 | 1.5 ± 0.2 | 2.0 ± 0.3 | 0.0 ± 0.0 | 0.0 ± 0.0 | 1.0 ± 0.1 | 2.5 ± 0.4 | 2.5 ± 0.4 | 1.0 ± 0.1 | 1.0 ± 0.1 |  | <0.001 | 0.323 |
| *Eubacterium* | 3.8 ± 2.5 | 4.7 ± 0.8 | 3.5 ± 0.7 | 6.5 ± 0.4 | 4.1 ± 0.8 | 6.3 ± 1.9 | 2.6 ± 1.0 | 3.0 ± 1.3 | 4.7 ± 1.2 | 6.5 ± 0.6 |  | <0.001 | 0.902 |
| *Faecalibacterium* | 4.5 ± 1.4 | 4.7 ± 1.1 | 4.0 ± 2.1 | 8.3 ± 2.7 | 6.1 ± 3.5 | 6.3 ± 2.3 | 12.8 ± 3.6 | 5.1 ± 2.1 | 6.6 ± 1.6 | 10.8 ± 1.2 |  | <0.001 | 0.513 |
| *Lachnospira* | 0.0 ± 0.0 | 2.8 ± 0.8 | 0.1 ± 0.1 | 1.1 ± 0.2 | 0.4 ± 0.1 | 0.9 ± 0.4 | 0.1 ± 0.0 | 0.7 ± 0.4 | 0.3 ± 0.3 | 0.3 ± 0.1 |  | <0.001 | 0.630 |
| *Megamonas* | 16.1 ± 8.1 | 0.0 ± 0.0 | 0.0 ± 0.0 | 0.0 ± 0.0 | 0.0 ± 0.0 | 0.0 ± 0.0 | 0.1 ± 0.1 | 11.2 ± 7.9 | 1.6 ± 1.6 | 0.0 ± 0.1 |  | <0.001 | 0.301 |
| *Megasphaera* | 3.2 ± 2.0 | 0.0 ± 0.0 | 0.0 ± 0.0 | − | − | 0.5 ± 0.1 | 0.0 ± 0.0 | 4.2 ± 3.4 | 0.0 ± 0.0 | 0.0 ± 0.0 |  | <0.001 | 0.046 |
| *Mitsuokella* | 0.0 ± 0.0 | 0.0 ± 0.0 | 0.0 ± 0.0 | − | − | 0.9 ± 0.6 | − | 0.0 ± 0.0 | 2.5 ± 2.4 | 0.0 ± 0.0 |  | <0.001 | 0.559 |
| *Oscillibacter* | 0.4 ± 0.3 | 0.1 ± 0.1 | 0.1 ± 0.1 | 1.3 ± 0.4 | 2.4 ± 1.1 | 0.4 ± 0.1 | 0.0 ± 0.0 | 0.3 ± 0.4 | 1.0 ± 0.7 | 0.4 ± 0.1 |  | <0.001 | 0.482 |
| *Phascolarctobacterium* | 0.6 ± 0.3 | 0.9 ± 0.1 | 0.0 ± 0.0 | 0.6 ± 0.1 | 1.5 ± 0.3 | 0.0 ± 0.0 | 1.8 ± 0.5 | 0.2 ± 0.3 | 0.4 ± 0.2 | 0.8 ± 0.2 |  | <0.001 | 0.631 |
| *Pseudoflavonifractor* | 0.1 ± 0.1 | 0.1 ± 0.0 | 0.0 ± 0.0 | 0.2 ± 0.1 | 0.4 ± 0.1 | 0.6 ± 0.2 | 0.0 ± 0.0 | 0.4 ± 0.2 | 0.2 ± 0.1 | 0.1 ± 0.0 |  | <0.001 | 0.999 |
| *Roseburia* | 0.1 ± 0.1 | 4.7 ± 1.2 | 0.2 ±0.2 | 3.7 ± 0.7 | 1.3 ± 1.1 | 1.1 ± 0.6 | 0.7 ± 0.2 | 1.5 ± 0.8 | 1.1 ± 0.6 | 0.8 ± 0.4 |  | <0.001 | 0.359 |
| *Ruminococcus* | 5.7 ± 1.9 | 5.9 ± 0.5 | 5.0 ± 2.0 | 7.2 ± 1.4 | 10 ± 1.4 | 10.4 ± 0.6 | 9.8 ± 2.0 | 3.4 ± 1.4 | 4.7 ± 0.9 | 9.7 ± 1.8 |  | <0.001 | 0.225 |
| *Sporobacter* | 0.0 ± 0.0 | − | 0.0 ± 0.0 | 0.2 ± 0.0 | 0.3 ± 0.1 | 0.2 ± 0.1 | 0.0 ± 0.0 | 0.0 ± 0.0 | 0.6 ± 0.5 | 0.0 ± 0.0 |  | <0.001 | 0.858 |
| *Streptococcus* | 2.7 ± 2.2 | 0.8 ± 0.3 | 12.9 ± 6.5 | 1.4 ± 0.4 | 1.2 ± 0.5 | 0.2 ± 0.1 | 0.6 ± 0.2 | 2.2 ± 1.2 | 0.5 ± 0.1 | 0.6 ± 0.3 |  | <0.001 | 0.881 |
| *Subdoligranulum* | 1.9 ± 0.7 | 2.9 ± 0.6 | 1.9 ± 0.6 | 0.1 ± 0.0 | 9.8 ± 0.5 | 2.7 ± 0.4 | 0.2 ± 0.1 | 2.1 ± 1.1 | 5.8 ± 3.1 | 2.5 ± 0.6 |  | <0.001 | 0.235 |
| *Veillonella* | 0.0 ± 0.0 | 0.0 ± 0.0 | 3.5 ± 1.5 | 0.0 ± 0.0 | 0.0 ± 0.0 | 0.0 ± 0.0 | 0.0 ± 0.0 | 0.1 ± 0.0 | 0.0 ± 0.0 | 0.0 ± 0.0 |  | <0.001 | 0.537 |
| Proteobacteria |  |  |  |  |  |  |  |  |  |  |  |  |  |
| *Brevundimonas* | 0.0 ± 0.0 | 0.4 ± 0.2 | 0.7 ± 0.7 | 0.2 ± 0.1 | 0.0 ± 0.0 | 0.1 ± 0.0 | 0.2 ± 0.2 | 1.0 ± 0.9 | 1.9 ± 2.6 | 4.7 ± 4.5 |  | <0.001 | 0.005 |
| *Mesorhizobium* | 0.0 ± 0.0 | 0.1 ± 0.0 | 0.2 ± 0.2 | 0.0 ± 0.0 | 0.0 ± 0.0 | 0.0 ± 0.0 | 0.1 ± 0.1 | 0.3 ± 0.3 | 0.2 ± 0.1 | 0.3 ± 0.2 |  | <0.001 | 0.052 |
| *Parasutterella* | 0.0 ± 0.0 | 1.6 ± 0.3 | 0.0 ± 0.0 | 0.0 ± 0.0 | 0.4 ± 0.1 | 0.4 ± 0.1 | − | 0.0 ± 0.0 | 0.0 ± 0.0 | 0.0 ± 0.0 |  | <0.001 | 0.290 |
| *Sutterella* | 1.1 ± 0.2 | 0.0 ± 0.0 | 0.0 ± 0.0 | 0.0 ± 0.0 | 0.6 ± 0.3 | 0.0 ± 0.0 | 0.0 ± 0.0 | 0.1 ± 0.2 | 0.2 ± 0.1 | 1.3 ± 0.4 |  | <0.001 | 0.220 |
| Verrucomicrobia |  |  |  |  |  |  |  |  |  |  |  |  |  |
| *Akkermansia* | 0.0 ± 0.0 | − | 0.0 ± 0.0 | 0.4 ± 0.1 | 8.1 ± 6.0 | 0.0 ± 0.0 | 0.0 ± 0.0 | 0.2 ± 0.6 | 0.1 ± 0.1 | 0.6 ± 0.6 |  | <0.001 | 0.930 |
| Others | 8.1 ± 2.0 | 9.7 ± 0.5 | 7.3 ± 1.7 | 28.5 ± 2.2 | 11.2 ± 1.1 | 31.5 ± 5.4 | 5.8 ± 1.0 | 9.2 ± 8.3 | 25.3 ± 10.9 | 9.4 ± 1.5 |  | <0.001 | 0.703 |

^a^ The individual means ± SD was calculated using all values for 1 week.

^b^ Based on Friedman test, the two variables “Subject” and “Day”, correspond to “inter-” and “intra-” individual variations, respectively.

^c^ Not detected.
